# Large-scale mapping of sequence-function relations in small regulatory RNAs reveals plasticity and modularity

**DOI:** 10.1093/nar/gku863

**Published:** 2014-09-27

**Authors:** Neil Peterman, Anat Lavi-Itzkovitz, Erel Levine

**Affiliations:** Department of Physics and FAS Center for Systems Biology, Harvard University, Cambridge, MA 02138, USA

## Abstract

Two decades into the genomics era the question of mapping sequence to function has evolved from identifying functional elements to characterizing their quantitative properties including, in particular, their specificity and efficiency. Here, we use a large-scale approach to establish a quantitative map between the sequence of a bacterial regulatory RNA and its efficiency in modulating the expression of its targets. Our approach generalizes the sort-seq method, introduced recently to analyze promoter sequences, in order to accurately quantify the efficiency of a large library of sequence variants. We focus on two small RNAs (sRNAs) in *E. coli*, DsrA and RyhB, and their regulation of both repressed and activated targets. In addition to precisely identifying functional elements in the sRNAs, our data establish quantitative relationships between structural and energetic features of the sRNAs and their regulatory activity, and characterize a large set of direct and indirect interactions between nucleotides. A core of these interactions supports a model where specificity can be enhanced by a rigid molecular structure. Both sRNAs exhibit a modular design with limited cross-interactions, dividing the requirements for structural stability and target binding among modules.

## INTRODUCTION

Bacterial small RNAs (sRNAs) are post-transcriptional regulators, typically non-coding and 50–250 base pairs long, involved in regulation of diverse biological functions in bacteria including metabolism, stress response, virulence and more (1–4). The best-studied sRNAs are encoded *in trans* with their target genes and perform their regulatory function by binding directly to the target mRNAs. In most known cases binding of the sRNA to its target leads to repression of gene expression by inhibition of translation or recruitment of a ribonuclease to degrade the mRNA (5–11). In other cases, sRNAs can activate their targets by inducing translation or by protecting the target transcripts from degradation (12–14). A single sRNA often acts specifically to regulate multiple targets. Many sRNA-target regulatory interactions require or are strengthened by the chaperone Hfq, a hexameric RNA-binding protein (15–19).

Much attention has been devoted to identifying sequence features involved in sRNA regulation (7,10,12–13,20–24). The relationship between sequence and function in sRNAs is important for identifying sRNA regulatory interactions from sequence data (25,26), engineering novel synthetic sRNAs (27,28) and understanding how sRNAs evolve (29). Typical sRNAs have conserved stem-loop structures including a GC-rich 3′ stem-loop that comprises, together with a downstream poly-U segment, a rho-independent transcriptional terminator. This structure facilitates a well-defined transcription termination site and protects the sRNA molecule from degradation (30,31). Many sRNAs also have a conserved A/U-rich Hfq binding site (16,32–33), but in some cases also require the terminator for recruitment of Hfq (34). Hfq-associated sRNAs feature *seed* sequences responsible for base pairing with a complementary region of their mRNA targets, most often located in its 5′UTR (12,35). The seed is critical to function and confers specificity for sRNA regulators; as little as a single nucleotide substitution interrupting base pairing in this region can abolish sRNA regulation (21,36). However little is known about how the different elements of an sRNA come together to define its ability to regulate its targets.

Sequence conservation analysis has proven useful in defining the seed region and predicting targets of bacterial sRNAs (7,37–38). However, this approach is restricted by the limited diversity in homologous genes that have been sequenced and can be identified. For example, sequence homologs of *ryhB*, an sRNA involved in iron homeostasis and stress response, have been identified (7) across Enterobacteriaceae as well as in other Gammaproteobacteria, and reveal a core sequence that is very highly conserved (Supplementary Figure S1A). This suggests that this core region is important for function but says little about other features and precludes a quantitative interpretation due to the rarity of sequence changes. Additionally, other features that are unique to a particular clade may be missed by a conservation-based approach.

Here we develop a systematic and unbiased strategy for precisely measuring the efficiency of a large library of sRNA variants to obtain a high-resolution quantitative mapping between sRNA sequence and function. Our approach, inspired by the recently developed sort-seq method (39–43), is applied to investigate two well-studied bacterial sRNAs. Unexpectedly, we find that the strength of the interaction can be tuned by changes to the seed sequence. Destabilizing peripheral structures is found to have direct—but not necessarily devastating—impact on the regulatory activity. Conversely, we show that destabilizing the structure around the seed may enhance interaction with non-specific targets. Our data suggest a modular structure of sRNAs, which we exploit to develop a quantitative model for predicting the efficiency of unmeasured variants. Possible implications of our findings on the evolution of sRNAs are discussed.

## MATERIALS AND METHODS

### Plasmids, strains and growth

All expression and abundance experiments were performed in BW-RI cells which were derived from *Escherichia coli* K-12 BW25113 (44), and constitutively express *tetR* and *lacI*, described previously (45). In experiments involving *ryhB* or variants of *ryhB*, a strain was used in which the endogenous *ryhB* gene was deleted from the chromosome, BW-RI Δ*ryhB*, and in one experiment a strain was used in which *hfq* was additionally deleted, BW-RI Δ*ryhB*Δ*hfq* (45). These strains were transformed by two plasmids, one encoding the target-reporter and the other containing the sRNA. The target gene 5′UTR and N-terminal codons fused to superfolder Green Fluorescent Protein (GFP) are expressed under the P_LlacO-1_ promoter, carried on an low-copy plasmid with the pSC101* ori (46,47). The sRNA and variants are expressed under the P_LtetO-1_ promoter and carried on a compatible p15A-based plasmid. Plasmid names, sources and cloning procedures can be found in Supplementary Text. For all expression and abundance experiments cells were grown in M63 minimal media, with 0.5% glucose and 0.1% casamino acids, under antibiotic selection with Carbenicillin and Chloramphenicol.

### Mutagenesis, sorting and sequencing

Libraries of sRNA variants were synthesized by random mutagenesis polymerase chain reaction (PCR; Agilent Genemorph II) performed on two templates, the endogenous *ryhB* and *dsrA* genes. These mutagenized amplicons were cloned into the sRNA expression vector by restriction and ligation, and transformed at high efficiency into 5-alpha electrocompetent cells (NEB). After growing overnight, the plasmid library was harvested and then transformed at high efficiency into expression strains already transformed with the target-reporter plasmid. Expression was then induced for 4 h in growth phase, and cells were sorted into 4–6 fluorescence levels using MoFlo Legacy Cell Sorter (Beckman Coulter). Gates were chosen evenly on a log scale in a range that includes about 90% of the cells in the library. The sRNA plasmid library was harvested from each bin and sequenced (Illumina). Mean fluorescence was inferred from read counts from each bin for unique variants using a maximum likelihood method. See Supplementary Text for further details.

### sRNA abundance measurements

sRNA variants listed in Supplementary Table S2 were synthesized using site-directed mutagenesis and transformed into expression strains. Cells were grown for 6 h under expression of each sRNA variant in absence of the target-reporter. At OD_600_ = 0.15–0.30, 300 μl cells were harvested and added to RNAProtect (Qiagen). RNA was purified using the miRNeasy Micro kit (Qiagen) with enzymatic disruption of the cell membrane. Reverse transcription of cDNA was performed with SuperScript III for reverse transcriptase-PCR (RT-PCR; Invitrogen). RT-PCR was performed on the cDNA with KAPA SYBR FAST Universal qPCR kit, measuring RyhB abundance and an internal control 16S (diluted 1000-fold due to its very high abundance). See Supplementary Text for further details.

### Additive model and interactions

As a null model for the effect of mutations on sRNA efficiency we define an additive model, where the contributions of different mutations are assumed to be independent. First, we use the qSortSeq assay to measure the fold-change *f_i_* in target expression due to sRNA variant *i* (as described in the Results section) and define this number as the *efficiency* of that variant. Next, we map a measured *f_i_* onto a modeled ‘energy’ function *S_i_* via $f_i = [1 + \exp ( - S_i )]^{ - 1}$. This functional form can be derived from a model where the sRNA-mRNA pair alternates between two states, active and repressed (derived in the Supplementary Text). From each measured variant *v* with a single mutation in which position *j* is substituted with nucleotide *σ* we obtain the ‘energy change’ due to each base mutation through $\Delta S_{j\sigma } = S_{\nu} - S_{{\rm WT}}$, where *S_ν_* is the energy measured for variant *ν* and *S*_WT_ is the energy measured for the wild-type sequence. If *σ* is the same as wild-type base at that position then $\Delta S_{j\sigma } = 0$.

The additive model then assumes that the energy of variants with multiple mutations is given by addition of the energy contributions of every mutation independently, such that $S_{{\rm add}} \left( {\sigma ^{(i)} } \right) = S_{{\rm WT}} + \sum\nolimits_j {\Delta S_{j\sigma _j^{(i)} } }$. Here, ***σ***^(*i*)^ is the sequence of variant *i* whose element $\sigma _j^{(i)}$ is the base at position *j*. The comparison between the measured *S_i_* and the predicted $S_{{\rm add}} \left( {\sigma ^{(i)} } \right)$ allows us to identify mutation interactions and estimate their strength. See Supplementary Text for details.

## RESULTS

### A quantitative high-throughput assay for sRNA efficiency

In order to probe the relationship between sequence and function in sRNAs, we sought to characterize the influence of small sequence perturbations on the ability of an sRNA to modulate the expression of its mRNA targets. We focused on two sRNAs endogenous to *E. coli*, RyhB and DsrA. RyhB is involved iron-stress response and maintenance of homeostasis through regulation of several target genes (48). Among these it represses *sodB* (7), which encodes an iron superoxide dismutase, and activates *shiA* (14), which encodes a shikimate permease. DsrA represses *hns* and activates *rpoS*, which respectively encode the histone-like nucleoid-structuring protein H-NS and the stress response sigma factor σ^S^ (12,13). Both sRNAs are well studied and have negatively and positively regulated targets, which allows us to compare the effect of mutations on both modes of regulation. The secondary structures of these sRNAs have been solved by combining RNase profiling with RNA secondary structure models (13,16), and are composed of three stem-loops, which we label SL1, SL2 and SL3 from 5′ to 3′ (Figure 2A and B). SL3 is part of the rho-independent transcription terminator in both sRNAs.

**Figure 1.**
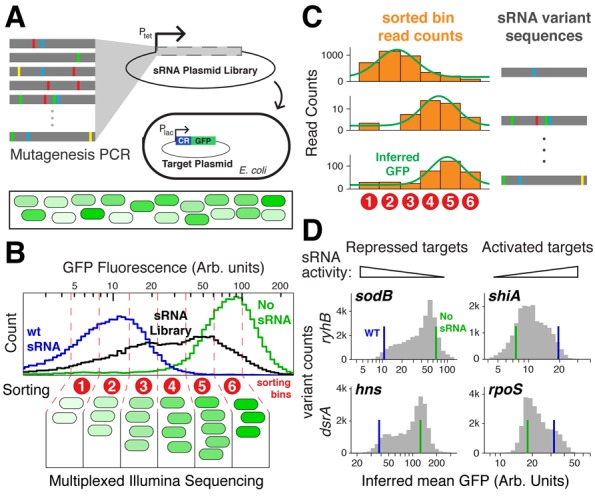
Large-scale mapping of sequence-function relation with qSortSeq. (A) Library generation: mutations are introduced to the sRNA gene using mutagenesis PCR. The resulting library is then ligated into a vector behind the *tet* promoter, and plasmids are transformed into an expression strain of *E. coli* carrying a compatible plasmid that expresses a target-reporter under a *lac* promoter. (B) Sorting and Sequencing: the transformed library is analyzed and sorted by flow cytometry. Cells carrying the transformed sRNA library (black) show fluorescence that spans the range between the wild-type sRNA (blue) to the empty-vector control (green). Plotted flow cytometry histograms are for RyhB repression of *sodB*. Cells are sorted into 4–6 bins in evenly spaced fluorescence intervals. DNA from each bin is purified, barcoded and sequenced (Illumina). (C) Analysis: for each sequence variant the number of reads corresponding to each sorting bin is tabluated (orange bars). The GFP fluorescence level is then inferred from the histogram of read counts using a maximum likelihood method. Examples from three variants from RyhB-*sodB* are shown. (D) Histograms of the 20–30 000 qSortSeq fluorescence measurements in each of the four measured libraries. For each target mean fluorescene with wild-type sRNA or no sRNA are highlighted in blue and green, respectively.

**Figure 2.**
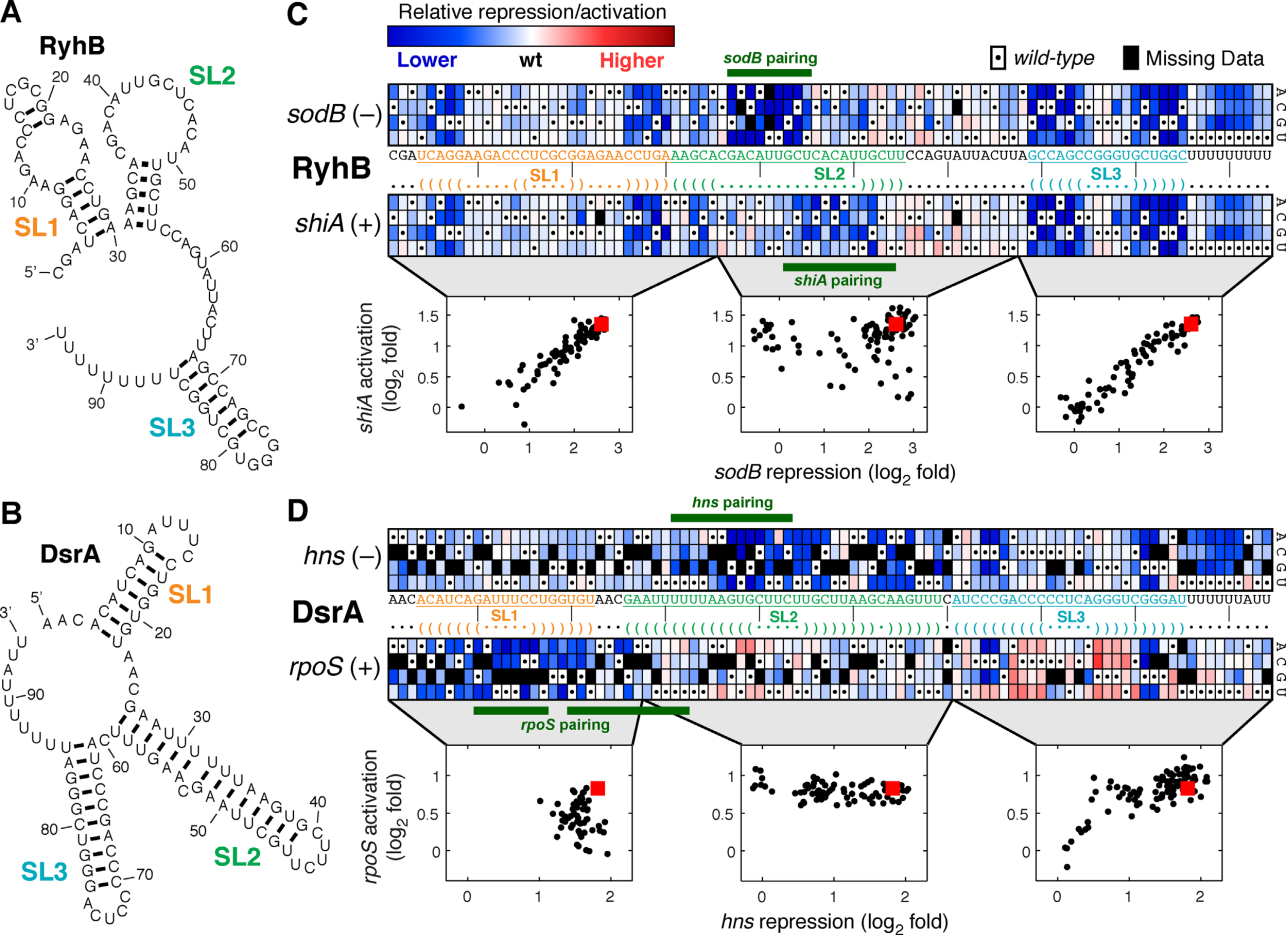
Effect of single mutations on sRNA efficiency. Known secondary structures of sRNAs tested, (A) RyhB (16) and (B) DsrA (13), with stem-loops annotated SL1, SL2 and SL3 from 5′ to 3′. (C) The effect of single nucleotide changes to RyhB on repression of *sodB* and activation of *shiA*. Each mutated sequence is represented by a box located at the position of the mutation and in a row that indicates the base at that position. Box color is scaled according to the sRNA efficiency (in logarithmic scale), normalized to the wild type. Black squares represent variants missing in the assay, and the wild-type base at each position is marked by a dot. Text denotes the wild-type sequence of the sRNA, along with the sRNA secondary structure, in which ‘•’ indicates an unpaired base, and ‘(‘or’)’ indicate downstream or upstream pairing, respectively. The region of the sRNA sequence that is complementary to each mRNA target is also annotated. Insets compare repression of *sodB* and activation of *shiA* by the RyhB variants, separated according to the position of the mutation (start, bases 1–35, Pearson's correlation *r* = 0.92, *N* = 104; middle, bases 36–67, *r* = 0.12, *N* = 94; and end, bases 68–94, *r* = 0.95, *N* = 78). (D) The effect of single nucleotide changes to DsrA on repression of *hns* and activation of *rpoS*, organized as above. Insets compare regulation of *hns* and *rpoS* expression (start, bases 1–27, *r* = −0.14, *N* = 58; middle, bases 28–60, *r* = −0.32, *N* = 72; and end, bases 61–94, *r* = 0.76, *N* = 91).

To quantify the efficiency of an sRNA with respect to any of its targets we measure fold-change in expression of the target upon sRNA expression using target-reporters containing the 5′UTR and several downstream nucleotides (in order to guarantee integrity of the local RNA structure), fused to the coding region of superfolder GFP. Both the sRNA and its target were placed behind synthetic promoters and carried on low-copy plasmids. These target-reporters are genetically different from the endogenous target mRNAs and, like the sRNA itself, are expressed at higher levels than would typically be reached under physiological conditions. However, previous studies have determined that GFP fusions can mimic the expression of the endogenous targets both for identifying or confirming sRNA target interactions (49) and for studying their quantitative characteristics (45). Thus, we take fold-change in expression of target-reporters to define the efficiency of the sRNA in regulating its targets.

For RyhB and DsrA, we constructed and measured sRNA efficiency for large-scale libraries of randomly mutated sRNA genes using a quantitative sort-seq approach (qSortSeq, Figure 1A–C). We synthesized a library of sRNA genes using mutagenesis PCR under conditions where sequence variants in the library carry on average 2–3 base changes compared to the endogenous gene. We then ligated these mutagenized DNA fragments into plasmids and transformed them into cells that carried a target-GFP reporter for one of the targets of the sRNA (Figure 1A). We grew the cells and sorted them into 4–6 bins using fluorescence activated cell sorting. We then performed multiplexed Illumina sequencing on the sRNA plasmid libraries purified from each bin.

Flow-cytometry measurements of fluorescent bacterial strains have considerable noise, even for clonal variants. For example, the blue and green lines of Figure 1B show histograms of large clonal populations that carry a wild-type or inactive small RNA, respectively. We therefore aimed to estimate the mean GFP fluorescence across a population of cells carrying each variant. First, we made sure that each variant is represented by a significant cell population (50–70 cells on average). These cells fall predominantly into a few adjacent bins, and we use the sequencing read count in each bin to estimate their proportions (Figure 1C). We then use a maximum likelihood approach to estimate from this coarse-grained histogram the average GFP fluorescence for the variant. Finally, we define the *efficiency* of the sRNA variant as the fold-change in target expression, estimated for activated targets by dividing this mean fluorescence with that of the control, and vice versa for repressed targets.

For each of the four sRNA-target pairs described above (RyhB-*sodB*, RyhB-*shiA*, DsrA-*hns* and DsrA-*rpoS*) we carried out qSortSeq and obtained measurements for 20 000–30 000 variants (Figure 1D). These variants include most possible variants with a single nucleotide change (79–83% for DsrA, 98–99% for RyhB), and a considerable fraction of the possible double mutants (14–31%). While most of the variants in our libraries carry three mutations and more, these represent only a miniscule fraction of those possible (as the number of possibilities grows exponentially with the number of mutations). Coverage data for each of the qSortSeq experiments is reported in Supplementary Table S1. The central barrier to higher coverage is bias in mutagenesis PCR, which heavily prefers certain base changes to others, in addition to sorting and sequencing throughput limits. Nonetheless, the assay produced measurements of bacterial sRNA regulation unprecedented in precision and scale.

In order to validate the qSortSeq method we isolated 8–10 variants from each mutant library and assayed them individually, alongside the wild-type and empty-vector controls. We compared these measurements to results from qSortSeq (Supplementary Figure S2). In all experiments we found a linear correlation between fluorescence measurements from the individual variants and the qSortSeq measurements, demonstrating that the latter is suitable for quantifying the efficiency of thousands of sRNA variants at once.

### Single nucleotide changes highlight structural and functional components

We first considered the variants in our library that carry a single nucleotide mutation compared with the native sRNA. The efficiency of these variants is depicted in Figure 2, along with annotations of sRNA secondary structure (13,16) and target-complementary match regions (7,12–14) as previously described in the literature. In a *de facto* validation of the method, one can observe a clear pattern in these defined regions of the sRNA that are expected to be most sensitive to single nucleotide changes. Moreover, the impact of different substitutions is highly correlated (Pearson's correlation for pairs of substitutions at the same position ranges from 0.64 to 0.79 for the four sRNA-target pairs measured). This correlation is consistent with the notion that individual bases in the wild-type sequence play a base-specific role that is interrupted by any base change.

Single point mutations in *ryhB* that affected sRNA efficiency were most prominent in the stems of SL1 and SL3 as well as in the base-pairing region of each target mRNA (Figure 2C). We first sought to discern sequence features that are target-specific from those that are not. We reasoned that the functionality of a sequence feature, where mutations have a highly correlated effect on the repression and activation of its targets, is likely to be target-independent. To find such features we looked for continuous sequence fragments where the effects of mutations on both targets are strongly correlated (Figure 2C inset). We found that the two ends of the sRNA represent such fragments: the region between bases 1 and 35, that includes SL1 and part of the middle stem-loop SL2 (Pearson's correlation *r* = 0.92), and the one between bases 68 and 94, that includes the rho-independent transcription terminator (*r* = 0.95). This suggests that the role of SL1 and SL3 in RyhB is primarily target-independent. We note in passing that the strong correlations between the two data sets, which were obtained in completely independent qSortSeq experiments, further demonstrates the robustness and precision of the assay. Conversely, efficiency for the two targets was poorly correlated in the middle region between these segments (*r* = 0.12), supporting the notion that this region contains target-specific elements (7).

We observed a comparable pattern among *dsrA* variants with a single substitution (Figure 2D), including areas of differential efficiency (both higher or lower than the wild-type) in SL3 and near the regions complementary to each target, *hns* and *rpoS*. As with *ryhB*, we compared efficiency with respect to the two targets in each of three regions (Figure 2D inset). In the regions that carry the seeds of *dsrA* the effects of mutations on regulating the two targets were weakly anti-correlated (bases 1–27 for *rpoS*, *r* = −0.14, and bases 28–60 for *hns*, *r* = −0.32), reflecting the non-overlapping regions of target-sensitivity. The effect of mutations in the terminator (bases 61–94) were strongly correlated (*r* = 0.76), as expected from its known target-independent function.

The poly-U at the 3′ end of the sRNA has been shown to be essential for Hfq binding (23,34), which is required for both RyhB and DsrA function. Interestingly, three sRNA-target pairs (RyhB-*sodB*, RyhB-*shiA* and DsrA-*hns*) were quite sensitive to mutations in the 3′ poly-U (Figure 2C and D). In contrast, DsrA activation of *rpoS* was far less sensitive to mutations in this region, consistent with the observation that DsrA-*rpoS* regulation can persist in the absence of Hfq when the sRNA is overexpressed (50). Our data are, therefore, consistent with these results and demonstrate that the function of the 3′ poly-U can be interrupted with a single base substitution.

As expected, mutations in the base-pairing regions of *ryhB* and its targets have a strong deleterious target-specific impact on regulation (Figure 2C). For *dsrA*, however, the regions of target-specific sensitivity and sRNA-mRNA complementarity overlap only partially (Figure 2D); mutations in some bases predicted to pair with the target have only minimal effect on sRNA efficiency, while mutation of other neighboring bases that are not complementary to the target have a stronger effect. This observation may be explained by the long pairing regions of DsrA and its target, which could make internal weak AU bonds dispensable while requiring a sequence context to facilitate sRNA-mRNA binding. In the next sections we explore these two aspects further.

### Binding energy between sRNA and target predicts the effect of mutations in the seed region

**Figure 3.**
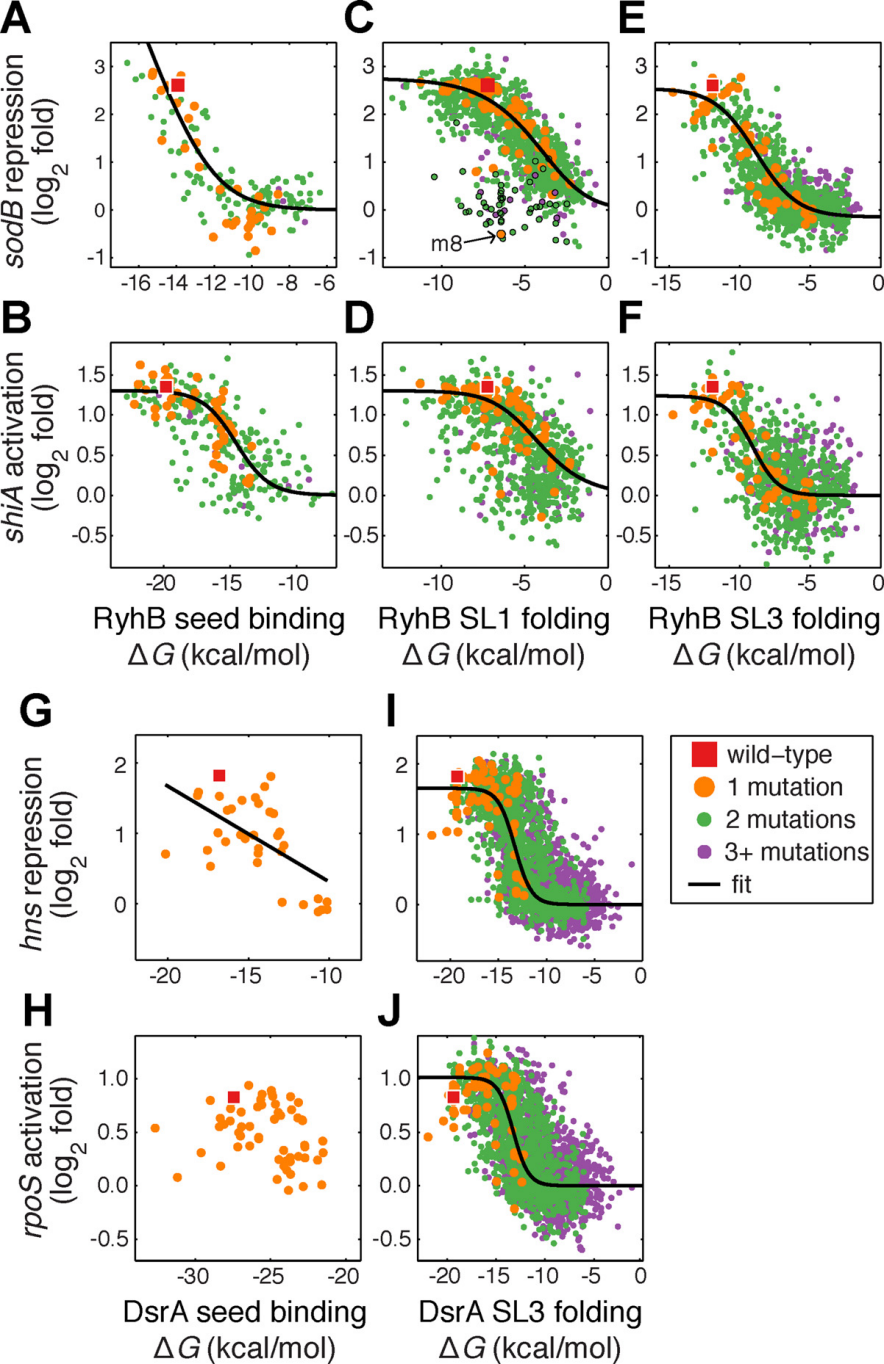
Effects of RNA binding energy and structure stability on sRNA efficiency. For each sequence variant we compare the change in local RNA stability or target binding free energy (kcal/mol) to sRNA repression/activation of its targets. (A–F) Variants of RyhB, (G–J) variants of DsrA. Seed interactions (A, B, G–H) are modeled as binding free energy of the sRNA-mRNA duplex. (A) RyhB-*sodB* (*R*^2^ = 0.62, *N* = 158), (B) RyhB-*shiA* (*R*^2^ = 0.42, *N* = 277), (G) DsrA-*hns* (*R*^2^ = 0.37, *N* = 35), (H) DsrA-*rpoS* (*N* = 60). Stem loop stability (C–F, I, J) is modeled by self-folding free energy. Stability of RyhB SL1 compared with regulation of (C) *sodB* (*R*^2^ = 0.46, *N* = 1223) and (D) *shiA* (*R*^2^ = 0.13, *N* = 792); stability of RyhB SL3 and regulation of (E) *sodB* (*R*^2^ = 0.57, *N* = 1089) and (F) *shiA* (*R*^2^ = 0.26, *N* = 908); stability of DsrA SL3 and regulation of (I) *hns* (*R*^2^ = 0.43, *N* = 2746) and (J) *rpoS* (*R*^2^ = 0.33, *N* = 2655). Variants carrying the mutation A30G are outlined with black circles in panel (C), and an arrow indicates m8, the variant that carries this mutation alone.

The effect of mutations on the free energy of sRNA-mRNA hybridization has been used previously to predict sRNA repression strength (21). We tested the relation between the free energy of the sRNAs-mRNA duplex and the measured efficiency of the sRNA variants in our libraries. For each variant that differs from the wild-type only in the seed region we computed target binding free energy Δ*G*_bind_ using the RNAcofold algorithm (51). We found that the computed binding free energy correlates strongly with the one estimated from the measured sRNA efficiency via a two-state binding model (see Materials and Methods; *R*^2^ = 0.62 for RyhB-*sodB*, Figure 3A; *R*^2^ = 0.42 for RyhB-*shiA*, Figure 3B; *R*^2^ = 0.37 for DsrA-hns, Figure 3G). For technical reasons we did not obtain variants with two point mutations in the seed regions of DsrA. Additionally, due to the length of the *rpoS* seed single point mutations do not greatly disrupt seed pairing or alter the computed free energy. As a result, the data pertaining to DsrA-*rpoS* seed pairing was limited (Figure 3H) and did not permit a reliable scrutiny of the relations between energy and efficiency.

We conclude that the target-binding free energy provides an excellent predictor for the effect of base changes in the vicinity of the sRNA seed. A long binding site is therefore more robust to such changes, while in shorter binding sites, however, base mismatches can be used to tune the response of a target to a small RNA. Indeed, in at least three of the cases we tested our libraries contained seed variants that covered the entire range of sRNA efficiency.

### Stable folding of stem-loops required for sRNA efficiency

As the effect of single mutations in the stems of stem-loop structures of each sRNA (particularly in SL1 and SL3 of RyhB and in SL3 of DsrA) was target independent, we hypothesized that the effect of these mutations is due to modulation of structural stability. To test this hypothesis we considered all variants with mutations only in one stem-loop structure, estimated the stability of their secondary structures by computing the self-folding free energy Δ*G*_fold_ of that stem-loop using RNAfold (51), and compared it with the measured sRNA efficiency (Figure 3C–F, I and J). In each case, the wild-type sRNA was at or near the maximum efficiency, and sequences that produce less stable folded structures (with higher ΔG_fold_) tended to result in lower efficiency, a relationship we fit with a logistic regression. The effect was similar for both positive and negative sRNA targets, despite the fact that the targets are regulated in different directions and through different mechanisms (8,13–14). We concluded that DsrA requires stable folding of SL3 in order to maintain sRNA efficiency, and that RyhB requires stable folding of both SL1 and SL3 for all targets tested. Furthermore, even small reductions in stem-loop stability can have (small) deleterious effects on sRNA efficiency.

In contrast to this general trend, the decrease in *sodB* repression efficiency of a few *ryhB* variants with mutations in SL1 was significantly greater than expected from this simple folding model (Figure 3C). Interestingly, most of these outliers (30 of 48) carry the transition A30G, a mutation to the most downstream nucleotide in SL1. It is therefore likely that this substitution has an additional effect on sodB regulation beyond weakening of SL1, an idea explored below.

### The effects of mutations on sRNA abundance and efficiency are not necessarily correlated

**Figure 4.**
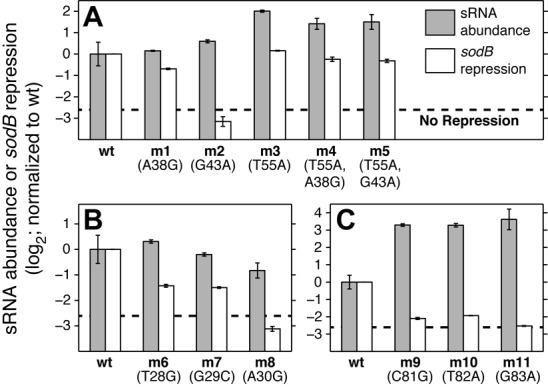
Changes in sRNA abundance due to mutations. sRNA abundance for RyhB variants, measured by qPCR in absence of its exogenous targets (gray bars), plotted relative to the wild type. Error bars are standard errors of the mean. qSortSeq measurements of the change in *sodB* repression (white bars). (A) Sequences carrying mutations in the seed region for *sodB* (m1, m2), and a mutation that recovers efficiency for these seed mutations (m3, m4, m5); (B) mutations in the 5′ stem-loop, SL1; (C) mutations in the 3′ stem-loop, SL3.

Our results, therefore, indicated that stem-loop stability is important for the efficiency of sRNAs in regulating their targets. What is the mechanism behind this? One possibility is that structural instability promotes mRNA degradation either directly or by interfering with transcription termination or Hfq recruitment. To explore this possibility we measured sRNA abundance by qPCR for several selected variants of *ryhB* with mutations in different regions of the gene (see Supplementary Table S2). We contended that abundance of the sRNA should reflect its destabilization as a balance between an unaltered transcription rate and a modified rate of turnover. These experiments were performed in absence of an expressed mRNA target-reporter so as to reflect changes to the sRNA itself and not the sRNA-target duplex or interaction. We plotted RyhB abundance relative to the wild-type alongside the change in efficiency in repressing *sodB* expression (Figure 4).

Two variants, each with a single mutation in the stem of SL1 (m6 and m7), which showed reduced repression of *sodB*, had similar abundance to the wild-type sRNA (Figure 4B). A third variant with one mutation in SL1 that eliminated repression of sodB (m8) showed only a 1.8-fold decrease in abundance. These examples suggest that the target-independent effects of mutations in SL1 are predominantly *not* due to overall increase in turnover of the sRNA.

Similarly, two variants with a single mutation in the seed region for *sodB* (m1 and m2), which showed a decreased or abolished repression of *sodB*, had similar abundances to the wild-type (Figure 4A), consistent with the idea that the effect of these mutations is due to weakened hybridization with the target. Conversely, a mutation at the base of SL2 (m3) resulted in a significant increase in abundance, possibly due to a change in the structure of RyhB SL2 that decreases the turnover of the molecule. When this mutation was combined with the mutations in the seed region (m1 and m2), repression of *sodB* was recovered and sRNA abundance was also increased (m4 and m5).

Based on these results we hypothesized that an increase in sRNA abundance can increase its efficiency. This hypothesis is supported by known properties of bacterial sRNA (45). To test this hypothesis we reasoned that a similar increase in efficiency should also be conferred by increasing the transcription rate of the sRNA. Indeed, changes in the level of induction of variant m1 increase its efficiency in regulating the expression of sodB (Supplementary Figure S4A). This, however, is not the only effect of the mutation in variant m3, as described below.

Contrary to our expectation, the abundance of sRNA variants with single mutations in the stem of SL3 (m9, m10 and m11), which all eliminated sRNA efficiency with respect to both targets, was 10- to 12-fold *higher* than the wild type (Figure 4C). Though Hfq is known to interact with the 3' stem-loop in some cases, this effect is *hfq*-independent (Supplementary Figure S5). As an alternative model, transcription termination failure, which can be caused by substitutions in the 3′ stem-loop (30), could lead to a more stable but inactive structure, resulting in higher sRNA abundance but abolished activity.

Together our results suggest that a plethora of mechanisms lead to changes in sRNA abundance. While some mechanisms may enhance the sRNA efficiency, as predicted by existing quantitative models of sRNA regulation, others may be accompanied by deleterious structural changes.

### Quantitative models balance required parameters and predictive power

Although a qSortSeq approach can yield the efficiency of a large number of sRNA variants, one can only ever expect to measure a small subset of all possible variants, since the number of possible mutants expands rapidly as the number of mutations increases. We therefore explored different approaches for constructing a quantitative predictive model of sRNA efficiency for arbitrary variants. Our large data set of variants with multiple mutations can be used to test the predictive power of these models.

**Figure 5.**
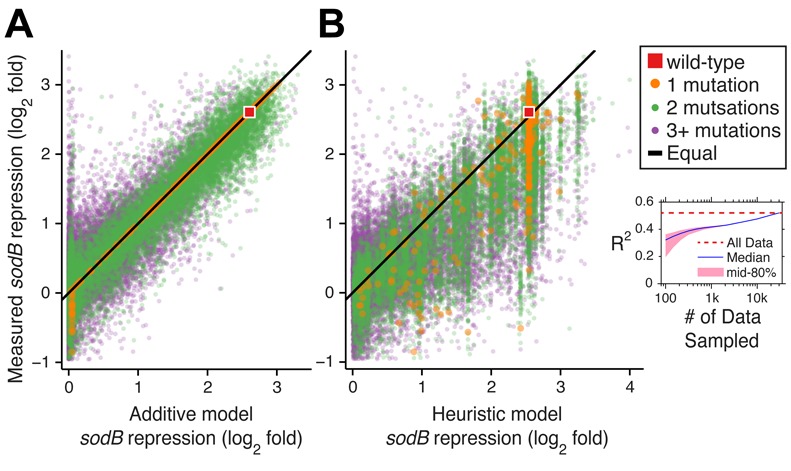
Quantitative models predict RyhB-*sodB* repression efficiency. Repression of *sodB* by all RyhB variants measured (*N* = 30 194) compared with predictions of (A) an additive model based only on measurements of single-mutation variants (*R*^2^ = 0.77) and (B) a heuristic model that incorporates folding and binding free energy predictions; a typical fit from 150 randomly sampled measurements (*R*^2^ = 0.35). (B, inset) *R*^2^ of the heuristic model fit with different numbers of randomly sampled measurements, with the median of 1000 trials in blue, the 10–90 percentile range in pink. For comparison the fit with all 30,194 qSortSeq measurements is indicated by the red dashed line.

First, we asked whether a simple additive model in which individual mutations give a fixed and independent contribution to the sRNA efficiency could be used to predict the efficiency of complex variants. We developed such a model based on a postulated two-state binding model in which a free energy function is taken to be the sum of single-nucleotide terms and can be written in terms of a position weight matrix. The 283 parameters for this model were obtained directly from qSortSeq measurements of single-mutation variants and the wild type. Model predictions were compared to measurements for RyhB-*sodB* (Figure 5A, *R*^2^ = 0.77 and 82% of predictions within 1.4-fold of measurements) and RyhB-*shiA* (Supplementary Figure S6, *R*^2^ = 0.33 and 79% of predictions within 1.4-fold of measurements).

One disadvantage of the additive model is that it requires measurements of all (or most) single mutation variants. We were therefore motivated to look for a heuristic model that requires a smaller number of measurements. In order to develop such a model we focused on the RyhB-*sodB* pair, and leveraged the relations established between sRNA efficiency and both sRNA-target binding (Figure 3A) and stem-loop structural stability (Figure 3C and E). Given an sRNA variant, we computed the folding free-energy of SL1 and SL3 and the binding-free energy between SL2 and the target. We then defined a heuristic energy function that combines the contributions of the three free-energy changes using six parameters that weight their relative contributions (see Supplementary Text for details). To demonstrate the predictive power of this model, we used just 150 measurements selected randomly from the library to fit the six model parameters (Figure 5B, interdecile range *R*^2^ = 0.28–0.38, with 61–64% of measured values within 1.4-fold of the predictions). Using more observation, the fitting power of this model could be increased up to *R*^2^ = 0.52 and 68% measured values within 1.4-fold of the prediction (Figure 5B inset).

The two alternative models we present make use of small subsets of measurements to map sRNA sequence with its efficiency. Despite their limited accuracy, both models do well in predicting trends in the data. The two models differ in two ways. First, the additive model requires measurements of nearly all single-mutation variants, while the heuristic model makes no demands on the type of measured variants. Second, the assumption behind the additive model—that *all* single mutations are independent—is stronger than those behind the heuristic model. This, however, is also an advantage of the additive model as deviations from its predictions indicate mutation interactions.

### Mutation interactions

In addition to predicting the efficiency of novel variants, a quantitative model can be used to highlight deviations from the assumptions of the model. We reasoned that variants that do not follow the additive model can reveal interactions between mutations, which can be used in turn to probe the functional organization of the molecule and identify functional modules. Nucleotides of the sRNA molecule act in concert to confer its structure and stability, to facilitate its interactions with Hfq and the mRNA target and more. We expected this cooperativity to be reflected in our assay as mutation interactions, also known as intramolecular epistasis (52). For example, two mutations may together reestablish a Watson–Crick pair that is disrupted by each one alone (synergistic interaction), or two mutations that have no effect individually may together have a deleterious effect (antagonistic interaction). Following this logic, we calculated the ratio between sRNA efficiency of each double mutant and the prediction of the additive model. A ratio that is significantly larger or smaller than 1 indicates an interaction between these two mutations, and we define the interaction strength (IS) as the log of this ratio. We call mutation interactions synergistic if the efficiency of the mutant is stronger (positive IS) and antagonistic if it is weaker (negative IS) than expected from the additive model.

**Figure 6.**
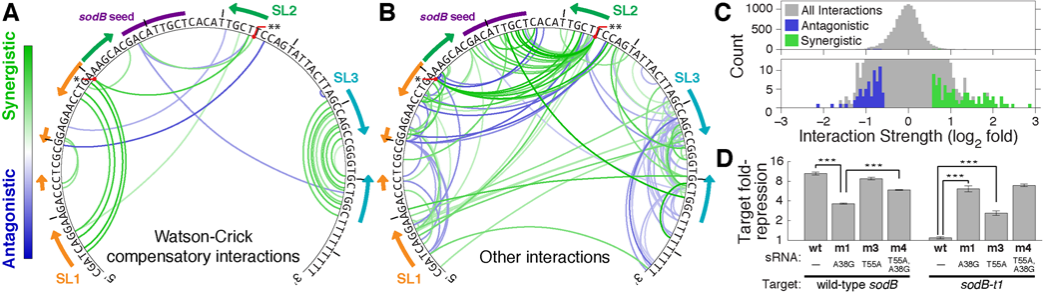
Mutation interactions for RyhB-*sodB*. Interactions are defined by deviations from the additive model for variants with two mutations compared to the wild-type. (A and B) Interaction maps for RyhB-*sodB*. The 150 most significant interactions (out of 11 910 pairs measured) are color-coded based on the strength and the ‘sign’ of the interaction, synergistic (green) or antagonistic (blue). RyhB sequence and the seed for *sodB* are annotated, and the secondary structure is marked by arrows indicating downstream and upstream pairing. The two single point mutations with the most interactions, A30G (*) and T55A (**), are marked. For clarity, compensatory interactions which maintain a potential Watson–Crick pairing lost by each mutation alone are plotted separately (A) from all other interactions (B). Eleven of the 21 compensatory interactions correspond to known stem-loop pairings in SL1 and SL3. (C) Histogram of IS, with mapped interactions highlighted in green (synergistic) and blue (antagonistic). (D) Seed-specificity assay: Fold-repression (log-scale) of two sodB variants, by wild-type RyhB and three sRNA variants, estimated from bulk GFP fluorescence measurements. ***P < 0.001 (two-tailed *t*-test).

For RyhB-*sodB*, our sRNA mutant library included data for 11 910 pairs of mutations, presented in Figure 6. Because the additive model provided a good approximation of repression efficiency for the vast majority of variants with two mutations (98% of measurements fall within 2-fold of the additive prediction), most pairs of mutations interacted weakly if at all. However, a small fraction of measurements showed strong interactions. Interactions that we consider both strong and statistically significant are presented in Figure 6A and B, and the distribution of all measured IS is plotted in Figure 6C.

We anticipated a clear signature of synergistic interactions due to the requirement of stable stem-loop structures for sRNA efficiency. In this case, two complementary mutations on both sides of a stem that individually interrupt its stability and therefore reduce efficiency can together recover stem-loop stability and thus sRNA efficiency, resulting in a strong synergistic interaction. All identified interactions that result from compensatory mutations (that is, pair mutations that restore potential Watson–Crick pairing lost by each mutation individually) are displayed separately (Figure 6A) from all others (Figure 6B). Such interactions were abundant within SL1 and SL3 in RyhB as expected, validating interaction-mapping approach. A zoomed-in interaction map for SL3 (Supplementary Figure S7) revealed many antagonistic interactions between bases in SL3 and the poly-U at the 3′ end of the sRNA, supporting the conclusion that these components operate together as a functional module (23). The strong mutation interactions were significantly enriched for interactions between two mutations within the same stem loop structure (85/150 interactions, *P* < 0.001 by Fisher's exact test). This result supports the model of modular functional organization, suggested above based on folding and binding free energies.

The interaction map draws further attention to two mutations that are involved in a large number of strong interactions (Figure 6B). One significant hub for mutation interactions across structural domains was A30G, a mutation at the base of SL1. Above we showed that this mutation had a stronger effect on the efficiency of RyhB than was expected from its effect on the computed folding free energy of SL1 (Figure 3C, black circles). As expected, this mutation had a strong interaction with its compensatory mutation in the stem of SL1. However, A30G additionally showed multiple synergistic interactions with nucleotides in the stem of SL2. This mutation potentially leads to a small structural change that extends the stem of SL2 (Figure 2A), and increases its structural stability. This could have a deleterious effect on efficiency if, for example, recognition or binding of the sRNA to its target requires structural flexibility (53,54) or involves opening the stem (55). In support of this model we note that A30G had multiple synergistic interactions with mutations in SL2 that loosen the stem-loop and could potentially reverse the stabilization effect of A30G.

Another highly interacting mutation was a transversion of the most downstream nucleotide in SL2, T55A. Above we showed that the variant that carries this mutation alone (m3) was slightly more efficient than the wild type and was significantly more abundant (Figure 4A), and rationalized that this increase in abundance contributed to its positive effect on two seed mutations. While it is possible that the stabilization effect of T55A is behind its positive effect on other deleterious mutations, as suggested above, this is unlikely to be the only effect since the mutations that interacted with T55A were focused in SL2 and found specifically in the seed region. Moreover, T55A was able to rescue variants with seed mutations that eliminated the regulation of the target completely, an effect that is unlikely to be due to an increase in abundance alone (Supplementary Figure S4B). Noting that the mutation T55A relaxes the stability of SL2, we hypothesized that this relaxed structure may be more susceptible to interaction with ‘off-targets’ that carry imperfect binding sites.

To test this hypothesis we constructed a target-reporter variant *sodB-t1* that carries a mutation in the seed region complementary to the RyhB mutation A38G. As expected, wild-type RyhB does not interact with this target, while variant m1, which carries the mutation A38G alone and therefore a perfect seed for *sodB-t1*, repressed it efficiently (Figure 6D). In support of our hypothesis, the mutation T55A allows the sRNA variant without A38G to repress *sodB-t1* significantly, despite the mismatched seed, but has no effect in the presence of a matched seed (compare m1 and m4 in Figure 6D).

**Figure 7.**
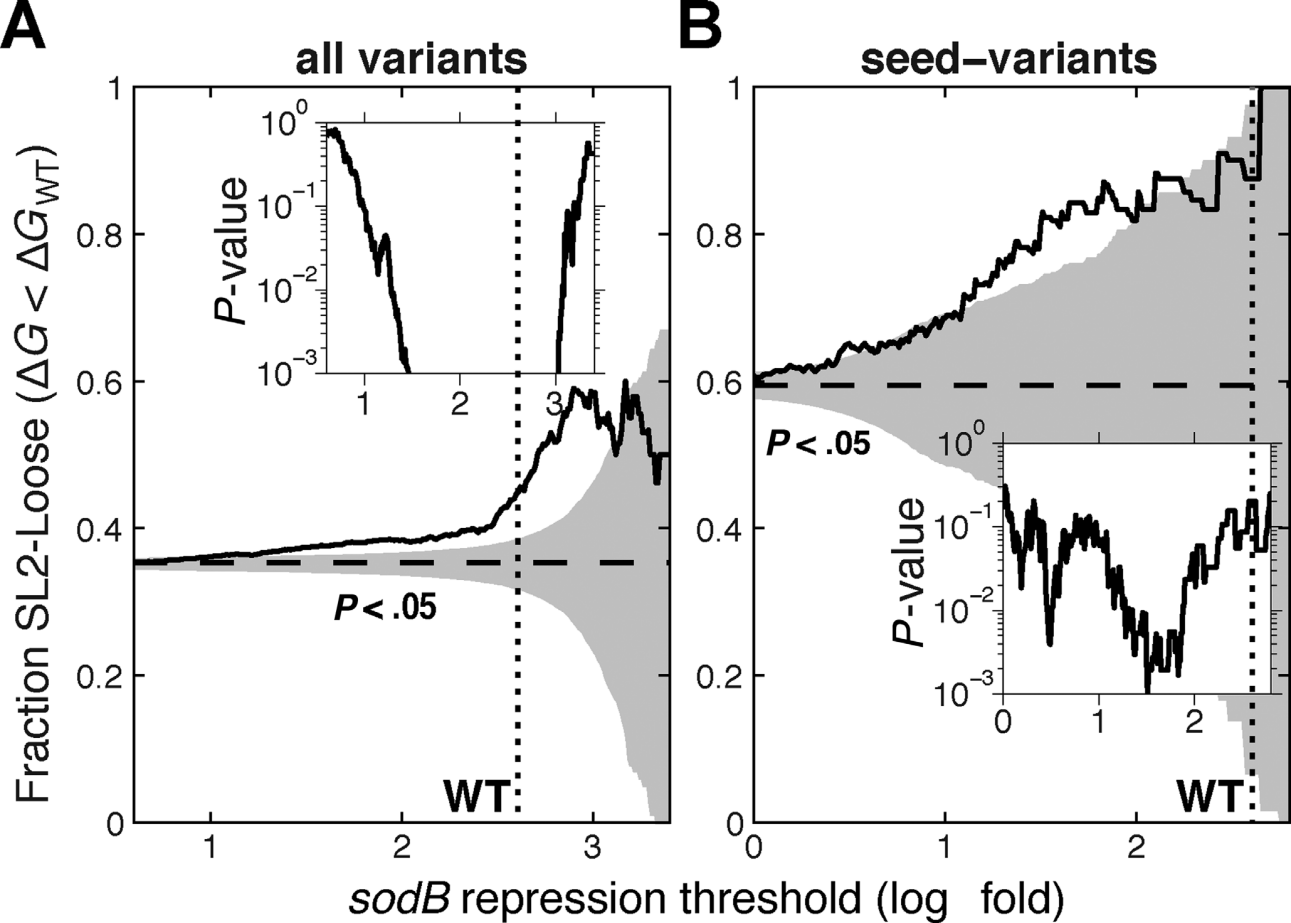
RyhB variants with high efficiency are enriched for loose structure in stem-loop 2. For a given repression level (horizontal axis) we ask what fraction of the variants that exhibit such repression or higher carry ‘loose’ SL2 (namely, stem loop whose folding free energy is greater than that of the wild type). Shaded areas correspond to enrichments that are not statistically significant (*P* > 0.05 by Fisher's exact test). The horizontal dashed lines indicate the fraction of variants with loose SL2 in the relevant data set, and the vertical dotted line indicates fold-repression of *sodB* by wild-type RyhB. Insets show the *P*-value at each efficiency level (*P*-value < 10^−3^ in the region where the curve is out of bounds). (A) Significant enrichment of variants with loose SL2 at intermediate and high repression strength is observed throughout the library (*N* = 30 194). (B) The fraction of loose SL2 is particularly high in variants that maintain efficiency despite a devastating mutation in the seed region (*N* = 4158).

Together, inspection of both mutation hubs supports the hypothesis that some flexibility in the structure of SL2 may be required for efficient binding, and that enhanced flexibility may relax some of the sRNA specificity. To test this hypothesis we examined the effect of SL2 structural stability on repression of *sodB* by other variants in the library. For each variant we calculated the self-folding free energy of SL2 using RNAfold (51). If this free energy was higher than that of the wild-type sRNA, we called the stem-loop of this variant ‘loose’. Indeed, we found that variants which manifested stronger repression were more likely to carry a loose SL2 (Figure 7A), and this effect became particularly significant for variants that exhibited repression similar to or stronger than the wild type. Moreover, variants that carried devastating mutations in the seed region but were recovered by additional mutations were highly likely to exhibit a loose SL2 (Figure 7B). In this case, the statistical significance assigned to this statement (inset of Figure 7B) was limited predominantly by the small size of the data set. This large-scale association corroborates the idea that some flexibility in the seed-carrying stem loop may enhance sRNA efficiency but lessen its specificity.

## DISCUSSION

The mapping between the sequence of a molecule and its function is complex, and depends strongly on its structure and kinetics, on the structure and abundance of other molecules and on cellular and environmental conditions. In turn, the sequence-function relation is the driving force of evolution, as mutated and recombined sequences are selected according to the ability of the molecule to perform its function. Obtaining a map between sequence and function—even at a coarse-grained level—is therefore essential for elucidating the evolutionary forces that act to shape the molecule. From a systems biology perspective, a map from sequence to function facilitates interpretation of the functional role particular molecules play within their respective systems.

Trans-encoded small regulatory RNAs, the focus of this study, are abundant control elements that act in pathways of diverse biological function. Here, we define the function of the sRNA as its ability to repress or activate each of its targets independently and draw quantitative links between these functions and the sRNA sequence and structure.

### qSortSeq reveals quantitative features of gene regulation

The function of a small RNA relies on structural stability and specific efficient interaction with its targets. We find that stability of the stem loop that harbors the seed region is not correlated with its efficiency (data not shown), and showed evidence that in some cases flexibility of the structure may be favorable. In contrast, maintenance of the structure of the other stem loops is required for efficient regulation. Our data suggest that the effect of stable folding on the efficiency of the small RNA can be modeled quantitatively as a two-state system. Two-state models for RNA hairpins are expected to hold for short stem-loop structures. Since those are abundant in small regulatory RNAs, we expect this result to hold for a large class of sRNAs.

Seed pairing between an sRNA and its target is determinant for the specificity of sRNA regulation. Our data expands on previous findings which suggested that perfect complementarity between the seed of RyhB and its *sodB* binding site is essential for regulation (21). The large scale of our data allowed us to observe functional seed sequences of partial complementarity. Such sequences differ from the wild-type sequence by a single GU wobble or by a mutation at the 3′ end of the seed. Interestingly, these are also the rules that define activity of eukaryotic microRNAs (56).

### Conservation and function

Many sRNA genes exhibit strong sequence conservation among identifiable homologs (Supplementary Figure S1A and B). This makes it difficult to use the statistics of conservation and co-conservation of sequence elements to infer function, and motivated us to develop qSortSeq as an alternative. Conversely, it is interesting to ask if the results of qSortSeq, which map sequence to function, can be used to interpret conservation patterns. We used the additive model to estimate the efficiency of homologous sRNA sequences (Supplementary Figure S1C), supposing that our results in *E. coli* are applicable to the efficiency of sRNAs in other species. Under these assumptions the model predicted that strong *sodB* repression is maintained in a number of species with divergent sequences. This result adds weight to the usefulness of the additive model, as most homologous sequences are farther in sequence space than most functional variants measured in the qSortSeq assay, and are operating in different cellular contexts. The availability of a simple predictive model bridges between high-throughput mutational assays and sequence conservation analysis, two powerful approaches to investigate sequence-function relations. We note in passing that genomic data highlights the importance of extending the qSortSeq approach to measure the effect of insertions and deletions and to incorporate them in a predictive model.

Mutations in SL1 of RyhB are found to have a wide range of effects on its efficiency, including some that completely abolish its effect on both targets. This result is surprising, since many bacterial species have RyhB homologs that do not have SL1 at all. Moreover, analysis of truncated sequences of *ryhB* (21), *dsrA* (12) and *sgrS* (57) suggests that the stem loop upstream of the one that harbors the seed is not necessary for repressing their respective targets.

A substantial fraction of the sequence variants in our library showed increased efficiency compared with the wild-type sequence (Figure 1D and Supplementary Figure S3). In RyhB many substitutions in the linker region between SL2 and SL3, known to mediate Hfq binding (16), increase its efficiency with respect to both tested targets. The efficiency of DsrA with respect to its targets is increased significantly by many mutations that weaken the stability of SL3, which in the wild type has a particularly strong GC-rich stem. As some of these sequence regions are highly conserved (Supplementary Figure S1A and B), it is likely that there are other trade-offs to these mutations, including, for example, the need to accommodate multiple targets. Above we demonstrated that greater target repression and sRNA stability can be associated with a decrease in regulatory specificity. Other possibilities, not explored here, include possible interplay with the translational machinery (A. Lavi-Itzkovitz *et al.*, under review) and the effect of mutations on the kinetics of regulation in caes when genes need to turned on or off rapidly, which could be critical for sRNAs that act in stress response pathways. While our method here focused on steady-state efficicency, the qSortSeq method can be adapted to study other properties, including kinetics and noise (N. Peterman and E. Levine, in preparation).

### Requirements for a predictive quantitative model

We presented a sort-seq approach tuned to obtain quantitative assessment and confidence intervals of sRNA efficiency. This was done simply by constructing the sequence library such that all sequences are represented in the library multiple times, by sorting to a small number of bins, and by postulating the shape of the underlying fluorescence distribution. This approach is particularly appropriate for studying sRNA sequence-function relations, since the efficiency of sRNAs is highly sensitive to mutations. A similar consideration may also be relevant for studying the 5′UTR of an sRNA target (42).

The modular structure of the sRNAs studied here suggests an attractive possibility of constructing a predictive quantitative model based on folding and target binding free energies. We showed that the efficiency of sRNA sequence variants could be predicted by a heuristic model that combined energy functions that characterize the effect of mutations in different regions, and uses a small subset of the data to fix its parameters. Thus, one can obtain a functional model even with data obtained from a qSortSeq assay with a relatively low coverage. This could, for example, facilitate multiplexing multiple sRNA species for an inexpensive sequencing reaction that would yield an approximate sequence-function map simultaneously for multiple sRNAs and target genes. Other possible generalizations may address the kinetics of sRNA regulation or competition among targets, both of which require an increase in sorting assays or conditions and could therefore be facilitated by a lower-depth approach. There may also be the potential to generalize the heuristic model to account for structural elements that move or shift in concert among conserved sequences. Such a model would also allow iterative parameter fitting in order to search more deeply through rapidly expanding phylogenetic data sets.

## SUPPLEMENTARY DATA

Supplementary Data are available at NAR Online.

SUPPLEMENTARY DATA
