# Supplementary material for: Large-scale mapping of sequence-function relations in small regulatory RNAs reveals plasticity and modularity
Source: Nucleic Acids Res. 2014 Sep 27;42(19):12177–88. doi: 10.1093/nar/gku863 (PMC4231740; doi:10.1093/nar/gku863)
Supplement: SUPPLEMENTARY DATA [file supp_42_19_12177__index.html]

Large-scale mapping of sequence-function relations in small regulatory RNAs reveals plasticity and modularity — Large-scale mapping of sequence-function relations in small regulatory RNAs reveals plasticity and modularity — SUPPLEMENTARY DATA 

# Large-scale mapping of sequence-function relations in small regulatory RNAs reveals plasticity and modularity

## SUPPLEMENTARY DATA

**Files in this Data Supplement:**

- SUPPLEMENTARY DATA
