# Supplementary material for: Large-scale mapping of sequence-function relations in small regulatory RNAs reveals plasticity and modularity
Source: Nucleic Acids Res. 2014 Sep 27;42(19):12177–88. doi: 10.1093/nar/gku863 (PMC4231740; doi:10.1093/nar/gku863)
Supplement: SUPPLEMENTARY DATA [file supp_gku863_nar-01604-h-2014-File009.pdf]

Large-scale mapping of sequence-function relations in small regulatory RNAs reveals plasticity and modularity: Supplementary Text

Neil Peterman, Anat Lavi-Itzkovitz and Erel Levine

Supplementary Materials and Methods

Plasmids

The plasmid pZE12S ($P_{Llac\text{-}O1}$:*crsodB-gfpmut3b*) was described previously [1]. *gfpmut3b* in pZE12S was replaced with *superfoldergfp* [2] by subcloning in the KpnI-XbaI sites to yield pZE12SF. $P_{Llac\text{-}O1}$:*crsodB-superfoldergfp* was then subcloned from pZE12SF into the XhoI-XbaI sites of pAS04 (a gift from P. Cluzel), a low-copy plasmid with the pSC101* ori [3], yielding pAS05, the target-reporter for *sodB*. 77 bases of the 5' Untranslated Region (5'UTR) and the first 46 codons of *shiA* were cloned from the chromosome of *E. coli* K-12 MG1655 into the EcoRI-KpnI sites of pZE12S to create pZE12A. *shiA* was then subcloned from pZE12A into pAS05 using XhoI-KpnI to create pAS06, the target-reporter for *shiA*. Target-reporters for *hns* and *rpoS*, pHns::gfp and pRpoS::gfp [4] (gifts from J. Vogel), were adapted to a system consistent with pAS05 and pAS06 (the plasmid backbone including *bla* and pSC101* ori, the promoter $P_{Llac\text{-}O1}$ and *superfoldergfp*). 36 bases from the 5'UTR and the first 28 codons of *hns* were cloned from pHns::gfp into EcoRI-KpnI to yield pAS07, the target-reporter for *hns*. 564 bases from the 5'UTR and the first 41 codons of *rpoS* were cloned from pRpoS::gfp and ligated into pAS05 using EcoRI-KpnI for the vector and a EcoRI-BsrGI for the PCR-amplified insert to create pAS08.

From the plasmid pZA31R ($P_{Ltet\text{-}O1}$:*ryhB*) [1], *ryhB* was replaced with a random sequence of DNA of equal length using its flanking NdeI-BamHI sites in order to create pZA31RF, an empty-vector control plasmid. From pZA31R, *ryhB* was also replaced with *dsrA*, taken from pBRdsrA [5] to create pZA31D.

sRNA mutagenesis

Random Mutagenesis PCR (Agilent Genemorph II) was performed on two templates, *ryhB* and *dsrA*, amplified from plasmids pZA31R and pZA31D, respectively. Primers used were CTA TCA GTG ATA GAG ATA CTG AGC ACA TAT GC and GAG CCT TTC GTT TTA TTT GAT GGA TCC AA. Mutagenesis was carried out in two rounds, the first amplifying 0.2 ng of template for 30 cycles, followed by a 1,000-fold dilution and a second round of another 30 cycles. The product was amplified again by one round of standard Taq PCR to increase yield. In all steps it was ensured that the largest dilutions left at least 10^7 DNA molecules in order to maintain library diversity. PCR products were digested sequentially with NdeI and BamHI and gel purified using a 2% agarose gel. A pZA31 vector backbone was prepared by sequential digestion of pZA31RF with NdeI and BamHI and was gel purified. 5 ligation reactions were carried out in parallel for each sRNA mutant library using T4 Ligase (NEB).

Ligated products were combined, purified and 4 µL was transformed into 100 µL of commercially prepared 5-alpha Electrocompetent *E. coli* (NEB). After 1 hour of recovery in 2 mL of the provided recovery media, a small fraction of the cells were plated in a 10-fold dilution series to measure the library's diversity, quantified by the number of independent transformants as measured by CFU. The transformations produced 2-4 million CFU. Isolated sRNA variants were sequenced for 16-20 transformants in order to estimate average mutation rate (2-3 mutations per variant) and validate the mutagenesis. The remaining cells were used to produce and store the sRNA plasmid library.

Subsequent analysis requires multiple sorted cells per sequence variant to make precise quantitative fluorescence measurements. In a library as diverse as this, many rare variants would not be measured frequently enough to allow for such a reliable estimate. In order to ensure that a subset of rare variants were sufficiently measured, the library diversity was truncated making some of these rare variants much more abundant and discarding others. This was achieved by culturing subsets of the library transformation directly after recovery. Fractions of the transformed cells representing between 0.3% to 50% were cultured separately in LB (with Cm) after the one-hour recovery. CFU measurements the following day indicated the culture that was originally inoculated by 100-200,000 viable transformed cells. This culture was aliquoted and frozen for long-term storage and miniprepped to purify the sRNA plasmid library. As a result of the

truncation, there are no more than 200,000 sequences in the library, though the number of unique variants in the library is likely much smaller due to common variants that independently appear more than once (e.g. variants with a single mutation).

Cell sorting and sequencing

Highly efficient electrocompetent cells (transformation efficiency $>10^6$ CFU/ng pure plasmid) were prepared using standard protocols from target-reporter strains. BW-RI + pAS07 and BW-RI + pAS08 cells were used to report efficiency of the *dsrA* library in regulating the targets *hns* and *rpoS*, respectively. BW-RI Δ*ryhB* + pAS05 and BW-RI Δ*ryhB* + pAS06 cells were used to report efficiency of the *ryhB* library in regulating the targets *sodB* and *shiA*, respectively. 10 ng of sRNA plasmid library was transformed into the these strains by electroporation and recovered in 1 mL SoC at 37°, shaking for 1 hour. A small fraction was plated in order to check CFU. The remainder was washed and diluted into 40 mL of minimal media shaken overnight at 37° C. Overnight cultures of individual strains with the target-reporter along with the wild-type sRNA or the empty vector were also prepared as controls. 12-16 hours later, cells from the library and the two control strains were washed twice and diluted 200:1 in 10 mL media. After 2-2.5 hours (when OD_{600} = 0.1-0.2), the samples were diluted to $OD_{600} = 5 \times 10^{-4}$ in 20 mL media with inducers (1 mM IPTG and 8 ng/mL aTc). After 4-4.5 hours of growth (when OD_{600} = 0.1-0.2), cells were put on ice and washed in PBS.

All flow cytometry analysis and Fluorescence Activated Cell Sorting (FACS) was done with the MoFlo Legacy Cell Sorter (Beckman Coulter) using a 70 µm nozzle and 60 psi pressure. Events were called using FSC and SSC thresholds, and fluorescence measurements were made with a 488 nm laser and 530nm/40 BW emission filter using peak height. Based on flow cytometry profiles of the library and two control strains, 4-6 gates were set for cell sorting such that the adjacent gate boundaries were evenly spaced (on a log-scale) and together spanned 80-90% of the cells in the library to be sorted (6 gates for RyhB-*sodB*, 5 for RyhB-*shiA*, 6 for DsrA-*hns*, and 4 for DsrA-*rpoS*). For each assay, approximately 2 million cells were sorted, two sort gates at a time, each for 25-30 minutes at a rate of 1,500-2,000 events per second. Sorting errors in which two cells were measured at once were found to increase dramatically with increasing event rate (unpublished data), though this protocol ensured a sort error rate of less than 1%. Cells were sorted into tubes on ice and aliquots of the library were changed every 15 minutes.

Sorted cells were added to LB with antibiotics, grown to saturation and miniprepped. The sRNA mutant genes were amplified with High-Fidelity PCR (NEB Q5) using PCR primers AAA CGG TGT AAC AAG GGT GAA C and CGT CAG ATT TCG TGA TGC TTG TC, starting with 10ng of plasmid DNA (including both the target plasmid and sRNA library plasmid) for 20 cycles. The amplicons were sequentially digested with NdeI and BamHI, and prepared for Illumina Sequencing using the TruSeq DNA Sample Preparation Kit (Illumina) with a modified protocol. The shearing step was skipped and a higher magnetic bead concentration was used in order to maintain the relatively short fragments in the purification steps [6]. Sorted samples were indexed and pooled proportionately to the number of cells sorted. The DNA was sequenced with Paired-End 100bp reads with HiSeq 2500 on two lanes. Due to low sequence diversity of the samples, the sequencing reaction was spiked with 40% PhiX DNA. Each DNA molecule was read in full forward and backward, providing a machine repeat. A stringent filter was applied to resulting data to decrease sequencing error rate: consensus between the forward and backward reads was required, and all bases in both reads are required to have $QS \geq 27$.

Inferring sRNA activity from sequencing data

Raw sequencing data r_{ij}, the number of reads of unique sequence i in sorted gate j, was processed using a maximum-likelihood approach to estimate the mean fluoresnce of each variant. The distribution of measured fluorescence values obtained in flow-cytometry experiments can typically be approximated by a log-normal distribution [7], such that $\mathbb{P}(x \mid \mu, \sigma) = \mathcal{N}_x(\mu, \sigma)$, where x is the log of fluorescence measured from a given cell and μ and σ are variant-specific mean and standard-deviation. Assuming that gate j corresponds to fluorescence values between l_j and u_j, the probability that a particular cell is sorted into gate j is given by

$$\mathbb{P}(j \mid \mu, \sigma) = \frac{\epsilon_j + (1-\epsilon) \int_{l_j}^{u_j} \mathcal{N}_x(\mu, \sigma) dx}{\epsilon + (1-\epsilon) \int_{l_{\min}}^{u_{\max}} \mathcal{N}_x(\mu, \sigma) dx} \; . \tag{1}$$

The parameter $\epsilon = 0.2$ was used empirically to account for any noise in the measurements, with $\epsilon = \sum_j \epsilon_j$ reflecting the relative likelihood of random sorts in each gate. The number of cells with sequence i sorted into bin j is estimated as $b_{ij} = d_j r_{ij}$, where d_j are proportionality constants that can be set by the entire dataset $d_j = \left(\sum_i b_{ij}\right) / \left(\sum_i r_{ij}\right)$. The

parameters μ_i and σ_i are estimated by maximizing their likelihood given the observed data,

$$\mathbb{P}(\mu_i, \sigma_i | \{r_{ij}\}) = \exp\left(\sum_j d_j r_{ij} \log\left(\mathbb{P}(j \mid \mu_i, \sigma_i)\right)\right) . \tag{2}$$

A normally distributed empirical prior was used based on the data from well-represented variants [8]. Error in these measurements was quantified using the observed Fisher information matrix [9]. The data was thresholded with number of sorts per unique sequence, requiring > 10 sorted cells per variant. Inferred fluorescence values that fell outside of the sorted range of fluorescence were excluded. Additionally, a small fraction of variants had inferred parameters indicating very low or high cell-to-cell variability. These variants typically had a low number of reads falling into a single gate or two highly divergent gates. Since these were likely to be unreliable estimates, they were also excluded based on very low or very high estimated σ_i, the parameter corresponding to cell-to-cell variability.

In order to quantify sRNA efficiency, target fluorescence in the presence of an sRNA variant is compared with bare fluorescence of the reporter in the absence of active sRNA. To estimate this value we used the mean fluorescence of variants with unstable SL3, which renders them inactive (Fig. 3*E*,*F*,*I*,*J*; $\Delta G_{\mathrm{SL3}} > -5$ kcal/mol for RyhB and $\Delta G_{\mathrm{SL3}} > -7$ kcal/mol for DsrA, see section below for details on computation of these values).

Verification measurements of isolated variants

In order to validate the qSortSeq measurements, 7-10 variants were isolated and assayed indiviually, along with wild-type and empty-vector controls. For RyhB, 10 variants were chosen and synthesized by site-directed mutagenesis of pZA31R, and transformed into the expression strains with target-reporters. For DsrA, colonies were selected and isolated directly from the transformed libraries and sequenced. Strains carrying the isolated variants (along with the wild-type and empty-vector control) were cultured overnight in minimal media, washed 2X in media and diluted 200-fold into 2 mL media and shaken at 37° C. After 2-2.5 hrs. (when OD_{600} = 0.1-0.2), cultures were diluted into 10 mL media with inducers (1mM IPTG, 8ng/mL aTc) to a concentration of $\mathrm{OD}_{600} = 5 \times 10^{-4}$. After 4-4.5 h of growth (when OD_{600} = 0.1-0.2), samples were taken and put on ice, washed in PBS, and analyzed by flow-cytometry (MoFlo, in the same configuration as with sorting above). GFP expression is the mean fluorescence of 50,000 events measurements, and is used to calculate sRNA activity.

sRNA abundance measurements

Variants listed in Table S2 were created by site-directed mutagenesis of pZA31R and transformed into BW-RI Δ*ryhB* along with pAS05. Overnight cultures of these strains and the wild-type were washed twice in media, diluted 200-fold and grown in 2 mL media for 2 hours. Absorbance was measured (OD_{600} = 0.03-0.05) and cells were diluted to $\mathrm{OD}_{600} = 5 \times 10^{-5}$ into 5 mL media with inducers (No IPTG, 8ng/mL aTc) in biological duplicates. Samples were incubated at 37° in a waterbath shaker. After 6 hours of growth, cells were still in exponential phase (OD_{600} is 0.15-0.3), 300 µL of bacterial culture was sampled and added to 600 µL RNAProtect Bacterial Reagent (Qiagen). RNA was extracted using the miRNeasy Micro kit (Qiagen) with enzymatic disruption of the cell membrane. Immediately following, reverse transcription of cDNA was performed with SuperScript III for RT-PCR (Invitrogen). RT-PCR was performed on the cDNA with KAPA SYBR FAST Universal qPCR kit, measuring RyhB abundance and an internal control 16S (diluted 1,000-fold due to its very high abundance), in two technical replicates. RT-PCR primers used were GAA GAC CCT CGC GGA GAA CC and CGG CTG GCT AAG TAA TAC TGG for ryhB, and AGG CCT TCG GGT TGT AAA GT and ATT CCG ATT AAC GCT TGC AC for 16S. RyhB abundance was measured relative to the 16S internal control measurement.

Bulk Fluorescence measurements

For two experiments, fold-change in gene expression was measured using a bulk fluorescence assay using strains with mutations in the sRNA and/or mRNA target reporter. These experiments and analysis of the data was carried out as in [10]. Briefly, cells were brought to steady state in 48-well plates in minimal media with inducers. Measurements of OD_{600} and GFP fluorescence were made between intervals of shaking at 37°. These quantities are related linearly, and the slope of the linear fit was taken to indicate average fluorescence of the strain. Fold-change is then measured by comparing these measurements with and sRNA or empty-vector control. For Figure 6*D*, this was carried out with the wild-type *sodB* reporter from pAS05 and a variant, *sodB-t1* which carries a mutation complementary to the mutation in *ryhB-m1* (A38G, see Table S2). The sequence of *sodB-t1* (from +1 to start codon) is ATACGCACAA TAAGGCTATT GTACGTATGC AAATTAATAA TAAAGGAGAG TAGCAATG**C**C ATTCGAATTA CCTGCACTAC CATATGCTGG

TACC, with the compensating mutation bolded and underlined. These experiments were carried out in biological triplicates.

Computation of RNA binding and folding free energy

RNAfold and RNAcofold, programs from the Vienna RNA Secondary Structure Package [11], were used to compute free energies for different components of the sRNA. In all cases ΔG, the difference in free energy between the folded and unfolded states of the RNA, is represented by ensemble free energy in order to account for the effect of sub-optimal configurations.

For sRNA-target base-pairing interactions, RNAcofold was used to compute the duplex free energy of the region of the seed of the wild-type sRNA and the region of the seed-match on the mRNA target. For RyhB-*sodB*, bases 35-48 of each ryhB variant was folded with bases 40-66 of the *sodB* reporter, (AUAAAGGAGA GUAGCAAUGU CAUUCGA). For RyhB-*shiA*, bases 41-58 of ryhB variants was folded with bases 11-39 of the *shiA* reporter, (AGAUCGACGG CAAUGUGAGU UACCUUUUC). For DsrA-*hns*, bases 29-48 of dsrA variants was paired with bases 33-65 of the *hns* reporter (UACAAUGAGC GAAGCACUUA AAAUUCUGAA CAA). For DsrA-*rpoS*, bases 1-26 of dsrA variants were matched with bases 436-478 of the *rpoS* reporter (CUUGCAUUUU GAAAUUCGUU ACAAGGGGAA AUCCGUAAAC CCG).

For sRNA stem-loop stability computations, RNAfold was used to report self-folding free energy of each stem-loop and 1-3 additional nucleotides on each end, indicating the reporting the folded free energy compared to unfolded. For RyhB, bases 1-31 were folded for SL1, bases 29-57 for SL2, and bases 67-88 for SL3. For DsrA SL3, bases 60-87 were folded. For all stem-loop free energy vs. activity plots, fold-change in target expression f is fit to ΔG with a logistic function,

$$f(\Delta G_{\text{SL}}) = 1 + \frac{c}{1 + \exp(-b(\Delta G_{\text{SL}} - a))} \tag{3}$$

where a, b, and c are fitting parameters.

The 2-state binding model

As discussed in the main text, the 2-state binding model is used to fit data from RyhB-*sodB* binding, and is also used in the development of quantitative models of sRNA regulation. Use of this simple model, used ubiquitously in the literature on Transcription Factor binding [12], can also be applied to RNA-RNA regulation. The model assumes a system with two states, bound and unbound, separated by free energy difference ΔE that accounts for binding affinity as well concentration of the molecule being bound. Statistical Mechanics determines the relative probabilities of the bound and unbound states with a Boltzmann factor $e^{-\beta \Delta E}$, where β is determined by temperature. Thus the probability of an unbound state, normalized by the two possible states, is

$$p_{\text{unbound}} = \frac{1}{1 + e^{-\beta \Delta E}} \ . \tag{4}$$

In the case of sRNA repression of an mRNA, the unbound mRNA is expressed normally whereas the bound molecules are not translated and are degraded. In absence of the sRNA, all mRNAs are unbound. Thus fold-change in target expression due to the sRNA is also indicated by the expression in equation (4). By relating ΔE to the sRNA-mRNA binding free energy, this is used to fit the effect of changes to RyhB-*sodB* binding

$$f(\Delta G_{\text{bind}}) = [1 + \exp(-b(\Delta G_{\text{bind}} - a))]^{-1} \tag{5}$$

where a and b are fitting parameters. This equation is also used to predict fold-repression from any sRNA sequence, in which $\beta \Delta E$ is replaced with an energy function S. Two such functions, corresponding the the additive model and the heuristic model, are derived below.

An alternative derivation

The above derivation of the 2-state binding model does not consider gene expression as a dynamical system, representing the constant expression and turnover of interacting molecules, but rather as a simple static 2-state system. Doing so requires additional complexity, but under a few assumptions will give similar results that are potentially more informative. The chemical kinetics of gene expression are considered, as has been used previously to analyze regulatory RNA [1, 13, 14]. Here, sRNA and mRNA are transcribed at rates α_s and α_m, and degraded at rates β_s and β_m, respectively. An sRNA and mRNA molecule form a duplex with rate k, which leads to their mutual degradation,

representing non-catalytic sRNA repression. The mRNA is translated with rate γ, and the resulting protein also degraded with rate β_p. The resulting chemical rate equations are taken at steady state, leaving the system of equations,

$$\begin{aligned} 0 &= \frac{\partial s}{\partial t} = \alpha_s - s\beta_s - (ms)k \\ 0 &= \frac{\partial m}{\partial t} = \alpha_m - m\beta_m - (ms)k \\ 0 &= \frac{\partial p}{\partial t} = m\gamma - p\beta_p \end{aligned} \tag{6}$$

with s, m and p indicating the concentration of unbound sRNA, unbound mRNA and protein, respectively. The steady-state solution gives $p = (\gamma/\beta_p)m$. In conditions in which the sRNA is much more abundant than the mRNA, the interaction with the mRNA only negligibly affects the steady-state sRNA concentration, giving $s \approx \alpha_s/\beta_s$. This assumption leads to the steady state-solution for m,

$$m = \frac{\alpha_m}{\beta_m + k\alpha_s/\beta_s} \; . \tag{7}$$

As a result the steady-state protein level, the concentration ultimately measured in a reporter-assay, is

$$p = \frac{\gamma\alpha_m/(\beta_m\beta_p)}{1 + k\alpha_s/(\beta_s\beta_m)} \; . \tag{8}$$

Removing the sRNA is equivalent to turning off its production, or setting $\alpha_s = 0$. Therefore by examining fold-change in target expression due to the sRNA, the terms in the numerator vanish leaving the simple expression,

$$f = \left[1 + \frac{k\alpha_s}{\beta_m\beta_s}\right]^{-1} \; . \tag{9}$$

If the parameters $k\alpha_s/\beta_m\beta_s$ are incorporated as a single Boltzmann factor, the result is exactly the same as with the more simple-minded derivation in equation (5). When only the sequence of the sRNA is altered, the constant β_m remains fixed as it represents degradation of the unaltered mRNA. Therefore in this picture, mutations that impact sRNA activity either impact the sRNA-mRNA interaction through changes to k or the expression and stability of the sRNA through α_s and β_s. This alternative derivation sheds light on the role of the free energy in the two-state binding in this system, and supports the applicability of this model in understanding sRNA regulation.

Two quantitative models for RNA regulation

An additive model was developed as described in Materials & Methods. Consider a variant i whose sequence is denoted by the vector $\boldsymbol{\sigma}^{(i)}$. Thus, for example, the wild-type sequence of *ryhB* (variant $i = 1$) corresponds to $\sigma_1^{(1)} = C, \sigma_2^{(1)} = G, \sigma_3^{(1)} = A$ etc. The fold-change in target expression is modeled as a function of the sRNA sequence $\boldsymbol{\sigma}^{(i)}$ using the two-state binding mode derived above,

$$f(\boldsymbol{\sigma}^{(i)}) = \left[1 + \exp\left(-S(\boldsymbol{\sigma}^{(i)})\right)\right]^{-1} \; . \tag{10}$$

The additive model is defined in terms of a Position-Weight-Matrix (PWM) $\Delta S_{j\sigma}$. Let variant ν be a single-mutation variant that carries the base σ at position j (that is, $\sigma_j^{(\nu)} = \sigma \neq \sigma_j^{(1)}$, but $\sigma_k^{(\nu)} = \sigma_k^{(1)}$ for any $k \neq j$). An entry in the PWM is the difference between the "energy"$S(\boldsymbol{\sigma}^{(\nu)})$ of this single-mutation variant and the energy $S_{\mathrm{WT}} \equiv S(\boldsymbol{\sigma}^{(1)})$ of the wild-type sequence itself:

$$\Delta S_{j\sigma} = S(\boldsymbol{\sigma}^{(\nu)}) - S_{\mathrm{WT}} \; . \tag{11}$$

Thus given the PWM we have the energy of any single-mutation variant: if this variant differs from the wild-type by base σ at position j, then its energy is $S_{\mathrm{WT}} + \Delta S_{j\sigma}$. The additive model assumes that the PWM can be used to calculate the energies of variants that carry multiple mutations by simply adding the PWM entries that correspond to each mutation. Thus, for example, if a variant carries two mutations, say σ_1 at position j_1 and σ_2 at position j_2, its energy in the additive model is given by $S_{\mathrm{add}} = S_{\mathrm{WT}} + \Delta S_{j_1\sigma_1} + \Delta S_{j_2\sigma_2}$. More generally for an arbitrary variant i we have

$$S_{\mathrm{add}}(\boldsymbol{\sigma}^{(i)}) = S_{\mathrm{WT}} + \sum_j \Delta S_{j\sigma_j^{(i)}} \; . \tag{12}$$

The parameters of the additive model, the entries $\{\Delta S_{jk}\}$ of the PWM, were thus fixed using the single-mutation variant data. Since the data contains some variants with $f(\boldsymbol{\sigma}^{(i)}) \leq 1$, a cutoff value is established ($f_{\text{cut}} = 1.01$), above which a cutoff energy is assigned ($\Delta S_{\text{cut}} = 5$).

In order to formulate a coarse-grained model for RyhB repression of *sodB*, a heuristic energy function S_{heur} was developed for the 2-state binding model, equation (10). S_{heur} has additive terms from each of the modules for seed-binding and self-folding of SL1 and SL3, which are themselves functions of ΔG_{bind}, ΔG_{SL1} and ΔG_{SL3}, values described in previous sections.

$$S_{\text{heur}} = F_1(\Delta G_{\text{bind}}) + F_2(\Delta G_{\text{SL1}}) + F_3(\Delta G_{\text{SL3}}) + \Gamma \tag{13}$$

Additive parameters from each of the from each of the three modular functions F_1, F_2 and F_3 are combined into Γ. The modular functions were devised by solving the energy function when the 2-state binding model is set equal to the model used to fit variants with mutations in a single module. The 2-state binding model itself was used to fit mutations in the seed in equation (5) resulting in simply

$$F_1(x) = \beta_1 x \tag{14}$$

For SL1 and SL3, a logistic regression was used, fitting the data with equation (3). Substituting parameters and removing the additive term results in

$$F_2(x) = \log\left(1 + \exp\left(\beta_2(x + \alpha_2)\right)\right) \tag{15}$$

and similarly for F_3. Substituting these equations into equation (12) results in

$$S_{\text{heur}} = \beta_1(\Delta G_{seed}) + \log\left(1 + \exp(\beta_2(\Delta G_{SL1} - \alpha_2)\right) + \log\left(1 + \exp(\beta_3(\Delta G_{SL3} - \alpha_3)\right) + \Gamma \tag{16}$$

which is the free energy function used to generate the heuristic model.

Mutation Interactions

In order to map mutation interactions the subset of variants with two mutations were examined. Within the additive model, equations (10) and (11), variant i with two mutations l and m is predicted to result in a fold-change

$$f_{\text{add}}^{(i)} = \frac{f_{\text{WT}}^{-1} - 1}{(f_{\text{WT}}^{-1} - 1) + (f_l^{-1} - 1)(f_m^{-1} - 1)}\,, \tag{17}$$

where f_{WT} is the fold-change of the wild-type sRNA and f_l and f_m are the fold-change of the variants each of the constitutive mutations. We then compare the measured fold-change for this variant, $f^{(i)}$, with the one predicted from the additive model, $f_{\text{add}}^{(i)}$. We define the interaction strength (IS) as

$$V^{(i)} = \log(f_i / f_{\text{add}}^{(i)})\,, \tag{18}$$

understood as deviations from the additive model. Negative values of V indicate synergistic interactions and positive values indicate antagonistic interactions.

References

[1] Levine E, Zhang Z, Kuhlman T, Hwa T (2007) Quantitative characteristics of gene regulation by small RNA. *PLoS Biol.* **5**:e229.

[2] Pédelacq JD, Cabantous S, Tran T, Terwilliger TC, Waldo GS (2006) Engineering and characterization of a superfolder green fluorescent protein. *Nat. Biotech.* **24**:79–88.

[3] Lutz R, Bujard H. (1997) Independent and tight regulation of transcriptional units in *Escherichia coli* via the LacR/O, the TetR/O and AraC/I1-I2 regulatory elements. *Nucleic Acids Res.* **25**:1203–1210.

[4] Urban JH, Vogel J. (2007) Translational control and target recognition by *Escherichia coli* small RNAs in vivo. *Nucleic Acids Res* **35**:1018–1037.

[5] Lease RA, Smith D, McDonough K, Belfort M. (2004) The small noncoding DsrA RNA is an acid resistance regulator in escherichia coli. *J. Bacteriol.* **186**:6179–6185.

[6] Rohland N, Reich D. (2012) Cost-effective, high-throughput DNA sequencing libraries for multiplexed target capture. *Genome Res.* **22**:939–946.

[7] Salman, H., Brenner, N., Tung, C., Elyahu, N., Stolovicki, E., Moore, L., Libchaber, A. and Braun, E. (2012) Universal protein fluctuations in populations of microorganisms. *Phys. Rev. Lett.* **108**:238105.

[8] Efron, B. (2010) Large Scale Inference Empirical Bayes Methods Estimation Testing And Prediction: Statistical theory and methods. Cambridge University Press, Cambridge, UK.

[9] Efron B, Hinkly DV (1978) Assessing the accuracy of the maximum likelihood estimator. *Biometrika* **65**:457-483.

[10] Lavi-Itzkovitz, A., Jost, D., Peterman, N. and Levine, E. (2014) Quantitative Effect of Target Translation on Small RNA Efficacy Reveals a Novel Mode of Interaction. *Under review.*

[11] Lorenz, R., Bernhart, S.H., Siederdissen, C.H. zu, Tafer, H., Flamm, C., Stadler, P.F. and Hofacker, I.L. (2011) ViennaRNA package 2.0. *Algorithms Mol. Biol.* **6**:26.

[12] Berg, O.G. and von Hippel, P.H. (1987) Selection of DNA binding sites by regulatory proteins: Statistical-mechanical theory and application to operators and promoters. *J. Mol. Biol.* **193**:723-743.

[13] Mehta, P., Goyal, S. and Wingreen, N.S. (2008) A quantitative comparison of sRNA-based and protein-based gene regulation. *Mol. Syst. Biol.* **4**:221.

[14] Mukherji, S., Ebert, M.S., Zheng, G.X.Y., Tsang, J.S., Sharp, P.A. and van Oudenaarden, A. (2011) MicroRNAs can generate thresholds in target gene expression *Nat. Genet.* **43**:854-859.

Supplementary Figure Captions

Fig. S1. Sequence alignments. Discontinuous Megablast alignment of *(A) ryhB* (245 matches) and *(B) dsrA* (147 matches). The endogenous sRNA sequence and secondary structure run along the top. Homologous sequence alignments are color-coded and aligned vertically. *(C)* The additive model applied to each homologous ryhB sequence in the search, ignoring missing or inserted bases.

Fig. S2. qSortSeq validation. Mean fluorescence per cell (Relative Fluorescence Units) for isolated variants plotted against qSortSeq maximum-likelihood estimates. Comparisons of log fluorescence for *(A)* RyhB-*sodB* (Pearson correlation $r = .96$, N=11), *(B)* RyhB-*shiA* ($r = .82$, N=11), *(C)* DsrA-*hns* ($r = .92$, N=8), and *(D)* DsrA-*rpoS* ($r = .48$, N=11).

Fig. S3. Mutations that increase repression/activation. For single nucleotide mutations that increase fold-repression or activation, we sum the change in log fold repression/activation for each nucleotide position. Stacked bar plots for *(A)* RyhB targets *sodB* and *shiA*, and *(B)* DsrA targets *hns* and *rpoS*. The sRNA sequence and secondary structure are included, as in Fig. 2.

Fig. S4. RyhB expression level and a seed mismatch. sRNA repression of *(A) sodB* and *(B) sodB-t1* is measured by bulk fluorescence for wild-type *ryhB* and variants carrying mutations A38G and/or T55A. All strains were measured with 5 ng/µL aTc, and strains with a seed mismatch and no T55A mutation (m1-*sodB* and wt-*sodB-t1*) were additionally measured at 8 ng/µL aTc. Error bars are SEM from six biological replicates. Mutation A38G changes an AU match between *ryhB* and *sodB* into a GU wobble, leading to about 30% reduction in efficiency. This reduction can be compensated by increasing the abundance of the sRNA *e.g.* by increased transcription. In contrast, the AC mismatch between wild-type *ryhB* and *sodB-t1* abolishes the interaction between the two molecules completely, which cannot be compensated by increased transcription of the sRNA alone. The partial recovery of regulation by mutation T55A confirms that the synergistic effect of this mutation extends beyond increasing the sRNA abundance.

Fig. S5. Effect of Hfq on sRNA abundance in RyhB SL3 variants. sRNA abundance for RyhB variants in an hfq^{-} background, plotted relative to the wild-type. Error-bars are standard errors of the mean. Variants each carry a single mutation, as in SL3 from Fig. 4*C*.

Fig. S6. RyhB-*shiA* additive model. Fold-activation of *shiA* for all RyhB variants measured (N = 22,373) compared with predictions of an additive model based only on measurements of single-mutation variants ($R^2 = 0.33$).

Fig. S7. RyhB-*sodB* SL3 interaction map. Less stringent thresholds result in 500 total interactions for the entire *ryhB* sequence. *(A-B)*. Interaction maps for a subset of RyhB including only the last 28 bases that comprise the rho-independent terminator, as in Fig. 5. 134 interactions fall in this region, *(A)* among them 16 compensatory pairs, and *(B)* other interactions, plotted separately. *(C)* Interactions plotted as a histogram.

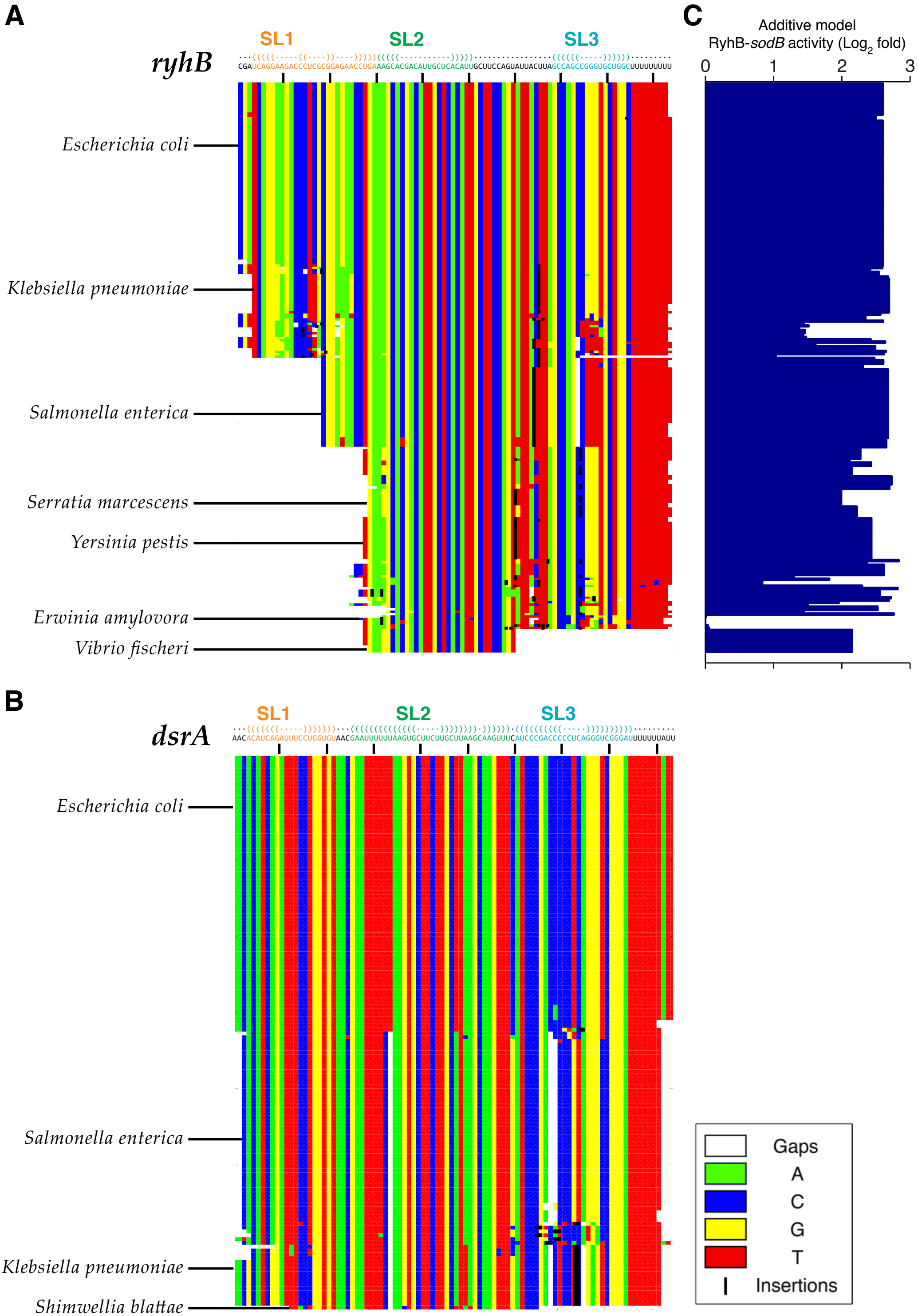

A
ryhB
SL1
SL2
SL3
CGAUCAGGAAGACCCUCGCGGAGAACCUGAAAGCACGACAUUGCUCACAUUGCUUCCAGUAUUACUUAGCCAGCCGGGUGCUGGCUUUUUUUU
Escherichia coli
Klebsiella pneumoniae
Salmonella enterica
Serratia marcescens
Yersinia pestis
Erwinia amylovora
Vibrio fischeri
C
Additive model
RyhB-sodB activity (Log2 fold)
0
1
2
3
B
dsrA
SL1
SL2
SL3
AACACAUCAGAUUUCCUGGUGUAACGAAUUUUUUAAGUGCUUCUUGCUUAAGCAAGUUUCAUCCCGACCCCCUCAGGGUCGGGAUUUUUUUAUU
Escherichia coli
Salmonella enterica
Klebsiella pneumoniae
Shimwellia blattae
Gaps
A
C
G
T
Insertions

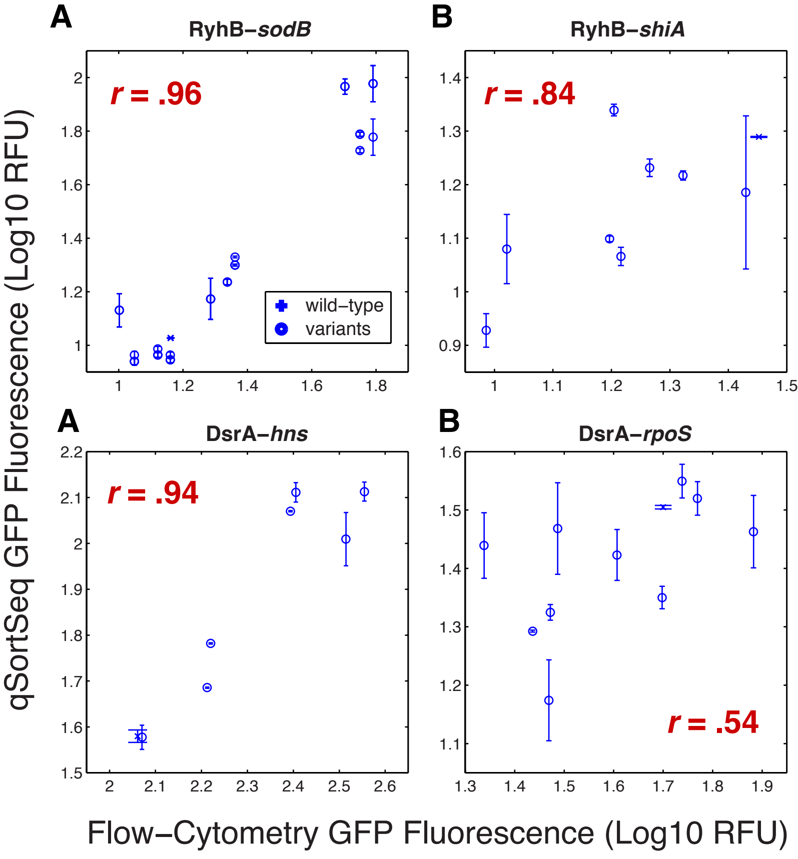
A
RyhB–sodB
r = .96
wild–type
variants
B
RyhB–shiA
r = .84
A
DsrA–hns
r = .94
B
DsrA–rpoS
r = .54
qSortSeq GFP Fluorescence (Log10 RFU)
Flow–Cytometry GFP Fluorescence (Log10 RFU)

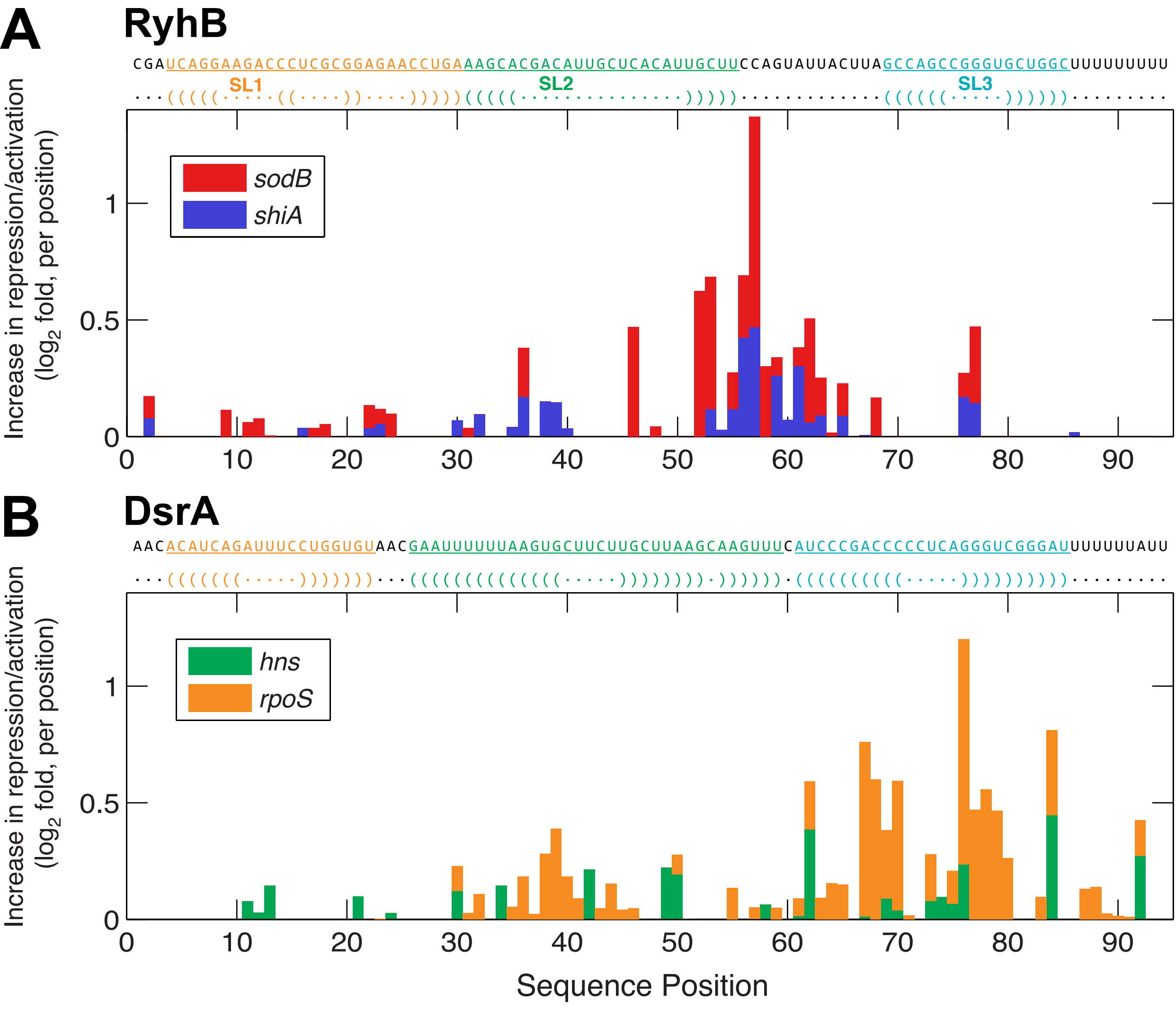

A
RyhB
CGAUCAGGAAGACCCUCGCGGAGAACCUGAAAGCACGACAUUGCUCACAUUGCUUCCAGUAUUACUUAGCCAGCCGGGUGCUGGCUUUUUUUUU
SL1
SL2
SL3
sodB
shiA
B
DsrA
AACACAUCAGAUUUCCUGGUGUAACGAAUUUUUUAAGUGCUUCUUGCUUAAGCAAGUUUCAUCCCGACCCCCUCAGGGUCGGGAUUUUUUUAUU
hns
rpoS
Increase in repression/activation
(log2 fold, per position)
Sequence Position

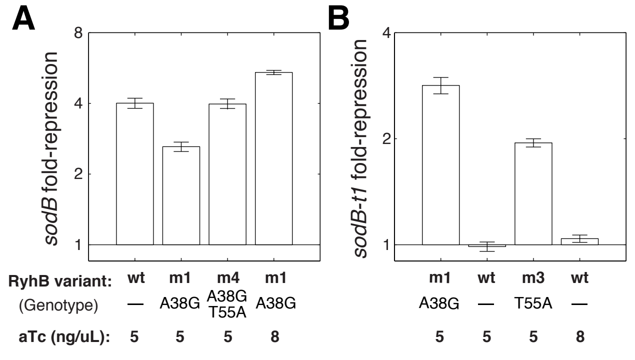

A
B
8
4
2
1
sodB fold-repression
4
2
1
sodB-t1 fold-repression
RyhB variant:
wt
m1
m4
m1
m1
wt
m3
wt
(Genotype)
—
A38G
A38G
T55A
A38G
A38G
—
T55A
—
aTc (ng/uL):
5
5
5
8
5
5
5
8

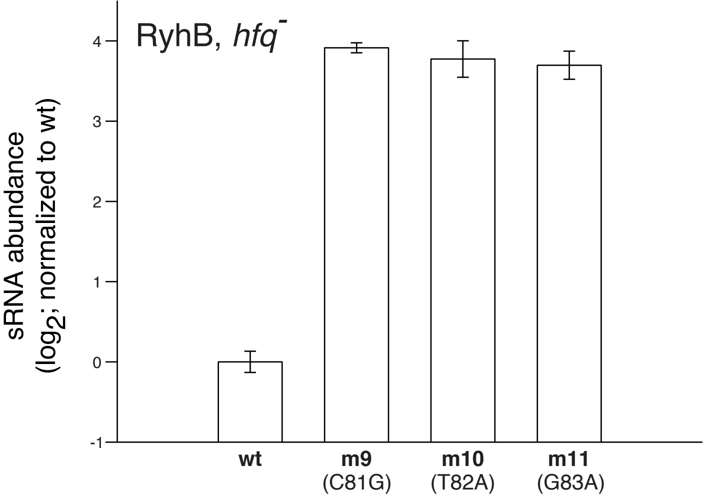

RyhB, hfq-
sRNA abundance
(log2; normalized to wt)
4
3
2
1
0
-1
wt
m9
(C81G)
m10
(T82A)
m11
(G83A)

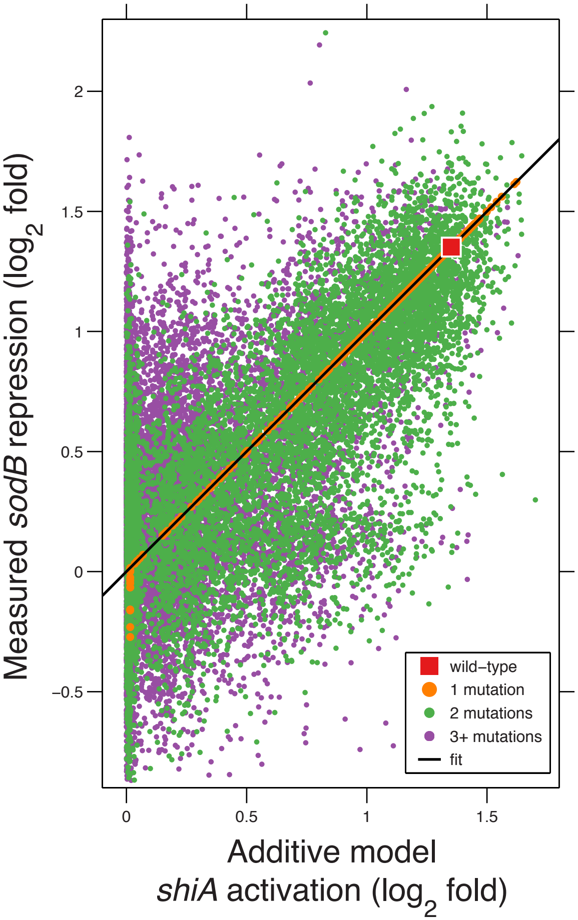

Measured sodB repression (log2 fold)
Additive model
shiA activation (log2 fold)
wild−type
1 mutation
2 mutations
3+ mutations
fit
−0.5
0
0.5
1
1.5
2

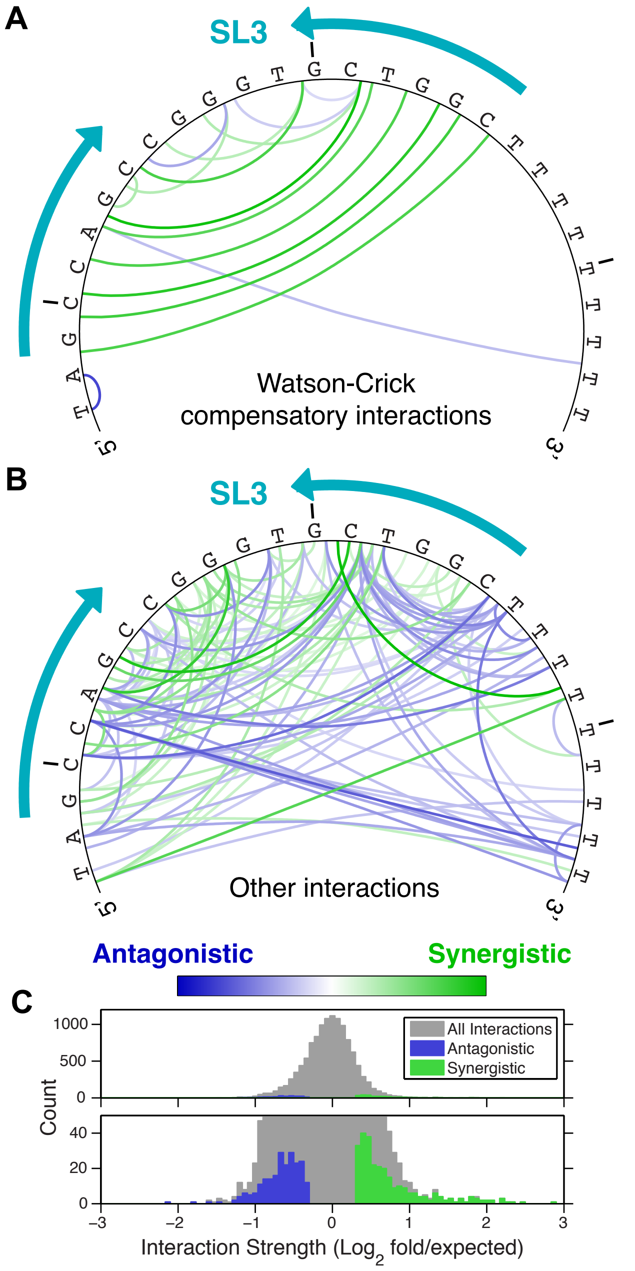
A
SL3
5'
3'
T A G C C A G C C G G G T G C T G G C T T T T T T T T T
Watson-Crick
compensatory interactions
B
SL3
Other interactions
Antagonistic
Synergistic
C
All Interactions
Antagonistic
Synergistic
Count
1000
500
0
40
20
0
–3
–2
–1
0
1
2
3
Interaction Strength (Log2 fold/expected)

Table S1: Coverage Statistics.

qSortSeq Assay	All variants	1 mutation (282 total)		2 mutations (39,339 total)		3 mutations (3,619k total)		4+ mutations
		N	%	N	%	N	%	N
RyhB-*sodB*	30,194	277	98.2	12,068	30.7	10,912	0.30	6,936
RyhB-*shiA*	22,373	280	99.3	9,066	23.0	7,662	0.21	5,364
DsrA-*hns*	28,031	223	79.1	5,739	14.6	13,265	0.37	6,082
DsrA-*rpoS*	27,435	233	82.6	5,586	14.2	13,154	0.36	5,898

Table S2: sRNA abundance, strains and measurements.

Mutant ID	Genotype	RyhB-*shiA* activation (qSortSeq, log_2 fold)	RyhB-*sodB* repression (qSortSeq, log_2 fold)	sRNA Abundance (qPCR, log_2 relative to wt)
**Experiment 1**				
wt	---	1.4	2.6	0.0 ± 0.6
***sodB* seed mutations**				
m1	A38G	1.2	1.9	+ 0.15 ± 0.02
m2	G43A	1.1	-0.5	+ 0.60 ± 0.07
**Recovery mutations**				
m3	T55A	1.4	2.8	+ 2.00 ± 0.05
m4	A38G, T55A	1.0	2.4	+ 1.4 ± 0.3
m5	G43A, T55A	1.3	2.3	+ 1.5 ± 0.3
**SL1 mutations**				
m6	T28G	0.6	1.2	+ 0.31 ± 0.06
m7	G29C	-0.3	0.9	- 0.20 ± 0.06
m8	A30G	0.0	-0.5	- 0.8 ± 0.3
**Experiment 2**				
wt	---	1.4	2.6	0.0 ± 0.4
**SL3 mutations**				
m9	C81G	0.1	0.5	+ 3.29 ± 0.08
m10	T82A	0.2	0.7	+ 3.3 ± 0.1
m11	G83A	0.0	0.1	+ 3.62 ± 0.06
